# Recent advances in user-friendly computational tools to engineer protein function

**DOI:** 10.1093/bib/bbaa150

**Published:** 2020-07-31

**Authors:** Carlos Eduardo Sequeiros-Borja, Bartłomiej Surpeta, Jan Brezovsky

**Affiliations:** 1 Laboratory of Biomolecular Interactions and Transport, Department of Gene Expression, Institute of Molecular Biology and Biotechnology, Faculty of Biology, Adam Mickiewicz University and the International Institute of Molecular and Cell Biology in Warsaw, Warsaw, Poland; 2 Laboratory of Biomolecular Interactions and Transport, Department of Gene Expression, Institute of Molecular Biology and Biotechnology, Faculty of Biology, Adam Mickiewicz University and the International Institute of Molecular and Cell Biology in Warsaw

**Keywords:** computational protein engineering, hotspot prediction, mutational analysis, semi-rational engineering, rational engineering

## Abstract

Progress in technology and algorithms throughout the past decade has transformed the field of protein design and engineering. Computational approaches have become well-engrained in the processes of tailoring proteins for various biotechnological applications. Many tools and methods are developed and upgraded each year to satisfy the increasing demands and challenges of protein engineering. To help protein engineers and bioinformaticians navigate this emerging wave of dedicated software, we have critically evaluated recent additions to the toolbox regarding their application for semi-rational and rational protein engineering. These newly developed tools identify and prioritize hotspots and analyze the effects of mutations for a variety of properties, comprising ligand binding, protein–protein and protein–nucleic acid interactions, and electrostatic potential. We also discuss notable progress to target elusive protein dynamics and associated properties like ligand-transport processes and allosteric communication. Finally, we discuss several challenges these tools face and provide our perspectives on the further development of readily applicable methods to guide protein engineering efforts.

## Introduction

Proteins have been studied intensively already for several decades to reap immense benefits through their applications in green industry, biomedicine, sustainable agriculture and other areas [1–7]. Prominent development of methods from genetic engineering and molecular biology has laid the foundation for protein engineering [8–10]. Initially, the main dilemma in protein engineering of which residues to target and which substitutions to introduce was approached either rationally or randomly. Rational engineering requires expert knowledge, often supported by increasingly available protein structures [11–13]. The random approach, dubbed directed evolution, mimics natural evolution by introducing a large number of mutations to be screened or selected [14,15]. Both engineering approaches profited extensively from progress in technology and scientific knowledge, enabling their systematic application, which led to numerous designs of proteins with improved function, solubility and stability. Their inherent requirements limit both methods. Directed evolution relies on a high-throughput system capable of evaluating large libraries of generated protein variants. In contrast, rational design requires a profound knowledge of the investigated protein and/or intensive computer simulations enabling the precise design of mutants.

**Table 1:** Computational tools for structure-based protein engineering

Target property	Tool	Distribution^a^	Obligatory inputs^b^	Outputs^c^	Section	Runtime^d^	Application status^e^	Link
Allostery	VisualCMAT [46]	WS	–	R, 3D, D	2.1	I / M	0 / 6	https://biokinet.belozersky.msu.ru/visualcmat
	PDB2Graph [47]	SA (L,W,M)	–	R, F	2.1	I / F	0 / 1	http://bioinf.modares.ac.ir/software/pdb2graph
	STRESS [48]	SA (L, M)	–	R	2.1	S / S	2 / 29	https://github.com/gersteinlab/STRESS
	AlloSigMA [49]	WS	–	R, F, 3D, D	2.1	E / E	13 / 36	http://allosigma.bii.a-star.edu.sg/home/
Protein–protein interactions	PPI3D [50]	WS	–	R, F, 3D, D	2.2	I / I	2 / 13	http://bioinformatics.ibt.lt/ppi3d/
	DisruPPI [51]	SA (L)	Interface region	N.A.	2.2	N.A.	0 / 3	N.A.^f^
	MutaBind [52]	WS	–	R, 3D, D	3.1	M / S	15 / 55	http://www.ncbi.nlm.nih.gov/projects/mutabind/
	iSEE [53]	SA (L, W, M)	Precomputed data^g^	R	3.1	I^h^	1 / 12	https://github.com/haddocking/iSee
	mCSM-PPI2 [54]	WS	–	R, F, 3D, D	3.1	I / M	1 / 7	http://biosig.unimelb.edu.au/mcsm_ppi2/
Protein–nucleic acid interactions	mCSM-NA [55]	WS	–	R, 3D, D	3.1	I / I	5 / 27	http://biosig.unimelb.edu.au/mcsm_na/prediction
	PremPDI [56]	WS	–	R, 3D, D	3.1	M / S	0 / 3	https://lilab.jysw.suda.edu.cn/research/PremPDI/
Protein–ligand interactions	mCSM-lig [57]	WS	Ligand affinity to wild-type	R, 3D	3.1	F / F	20 / 48	http://biosig.unimelb.edu.au/mcsm_lig/prediction
Ligand transport	CaverDock [58–60]	WS	Starting point of tunnels, ligand	R, F, 3D, D	2.3	F / F	4 / 9	https://loschmidt.chemi.muni.cz/caverweb/
Dynamics	DynaMut [61]	WS	–	R, F, 3D, D	3.2	F / M	43 / 68	http://biosig.unimelb.edu.au/dynamut/
Electrostatics	Mutantelec [62]	WS	–	R, F, 3D, D	3.3	M / S	2 / 2	https://structuralbio.utalca.cl/mutantelec/
	AESOP [63]	SA (L, W, M) WS	–	R, F, 3D	3.3	I / F	6 / 6	https://github.com/BioMoDeL/aesop/, https://aeolus.engr.ucr.edu/aesop/^i^
Complete pipeline	HotSpot Wizard [64,65]	WS	–	R, F, 3D, D	4	F / F	21 / 60	https://loschmidt.chemi.muni.cz/hotspotwizard/
Data integration	BioStructMap [66]	SA (L, W, M) WS	–	R, F	3.4	I/ F	0 / 0	https://github.com/andrewguy/biostructmap, https://biostructmap.burnet.edu.au/^i^

^a^WS, web-server and SA, standalone. For standalone tools, supported operating systems are listed: W, windows; L, linux and M, MacOS.

^b^All tools require structural input for the wild type (WT) protein or complex as PDB file or PDB id code, except for PPI3D and HotSpot Wizard, which alternatively can start from a protein sequence only.

^c^Formats of provided outputs: R, raw data; F, figures; 3D, 3D structure and D, downloadable data.

^d^Approximate calculation runtimes for small/large proteins: I, instantaneous (≤1 min); F, fast (≤5 min); M, moderate (6–15 min); S, slow (16–60 min); E, extensive (h); for details, see Supplementary Table 1 available online at https: //academic.oup.com/bib.

^e^Utilization of the tools are represented as the number of citations to the practical use of the tool/the total number of citations; for details, see Supplementary Table 2 available online at https: //academic.oup.com/bib.

^f^Contact authors (cbk@cs.dartmouth.edu).

^g^Inputs comprise the 3D structure of the WT and mutant complexes, eight energy terms, and evolutionary information.

^h^The runtime is reported for an example case for which non-trivial input data have already been precomputed.

^i^Web-page not accessible at the time of submission; N.A., not available.

The recent trend is to use both these approaches in unison, which is termed semi-rational engineering or focused directed evolution, to overcome the primary restrictions of the two approaches. In this strategy, rational components and computer predictions are used to prioritize the most promising protein sites for mutagenesis and frequently also implement restrictions on the diversity of introduced mutations to the most viable ones [16–21], resulting in smart-and-small mutant libraries with a large fraction of functional variants. With the tremendous advances in computer technology, availability of protein structure models and mathematical methods, computational tools have become indispensable for the semi-rational engineering process [22–26]. Such approaches continuously support the successful delivery of proteins adopted for use in various biotechnologies [27–32]. Following continuous efforts to introduce increasingly sophisticated computational approaches into semi-rational engineering, a plethora of new tools with distinct purposes and uses are being developed and released each year. This unceasing addition of available tools has positive and negative implications. On one hand, there is a tool available for almost any particular task; on the other hand, the resulting diversity of offered tools can be overwhelming for new users and active practitioners alike.

**Figure 1:**
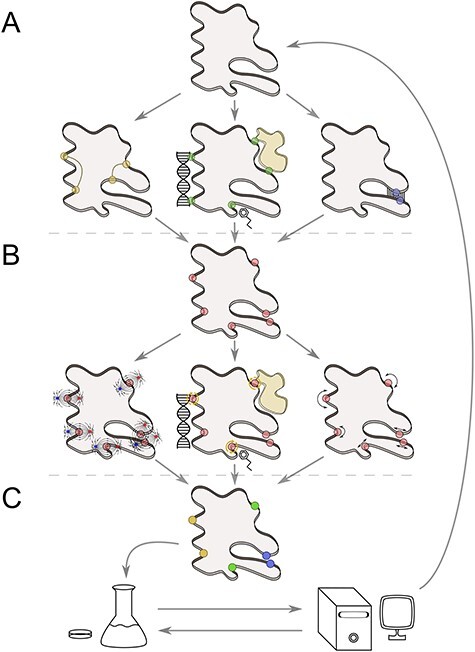
Overall engineering workflow employing the recently introduced user-friendly tools. (**A**) Using the structure of target protein (gray cross-sections of a protein body), the hotspots (shown as colored spheres) are identified, focusing on residues capable of cross-talk (yellow spheres), protein interactions with other (macro)molecules (green spheres) and ligand-transport tunnels (blue spheres). (**B**) Mutations in hotspots (red spheres) can be assessed by examining their effects on electrostatics (left), intermolecular interactions (center) or dynamics (right) to yield the best mutant candidates for experimental characterization. (**C**) A comprehensive analysis of experimental results for mutations at prioritized hotspots targeting various properties (colored spheres) can provide feedback to guide further engineering iterations.

To limit the never-ending literature search and guide researchers toward appropriate tools, we have critically surveyed structure-based computational tools dedicated to protein engineering that have emerged between 2016 and 2019 (Table 1). Tools published before this period have already been thoroughly reviewed [33–36]. We have focused on recent additions to the software toolkit of user-friendly and readily applicable approaches for altering protein function that can be employed by a broad spectrum of researchers, whereas more advanced tools and methods for protein engineering and design relying on the utilization of intensive computation and expertise have been reviewed elsewhere [37–43]. Also, we have not covered tools for the evaluation of protein stability or solubility, as those have been reviewed too [44,45]. As bioengineering research can comprise various strategies leading to the selection of the most promising candidates with improved function of interest, we consider tools for the following sequential stages of the engineering process: (i) hotspot identification for site-saturation mutagenesis (Figure 1A), (ii) *in silico* mutagenesis to evaluate the effects of mutations and prioritize promising variants (Figure 1B) and (iii) analysis of results to guide further engineering efforts (Figure 1C). Additionally, we discuss an integrative computational workflow that aims at providing complete computational support for protein engineers.

## Tools for hotspot identification

The initial step in a bioengineering project is to identify promising and relevant positions or regions of the protein to mutate. These sites, hotspots, are often located in or near structurally or functionally important regions of the protein, increasing the likelihood that mutations at these positions impact the protein’s properties. Various strategies utilizing protein structure for hotspot identification can be applied depending on the respective target property, e.g. focusing on the proximity of binding sites [67], ligand-transport pathways [68], flexible regions [69], allosteric networks [70] or conversely structural voids [71]. Nonetheless, the majority of such tools rely on an analysis of a multiple sequence alignment (MSA) to obtain insights into the importance of the individual positions in homologous proteins. Recently developed tools such as visualCMAT [46], PDB2Graph [47], STRESS [48] and AlloSigMA [49] aim to tackle the challenging prediction of hotspots involved in allosteric communication and others, including PPI3D [50] and DisruPPI [51], focus on hotspots which govern protein–protein interactions.

### Allosteric hotspots

**Figure 2:**
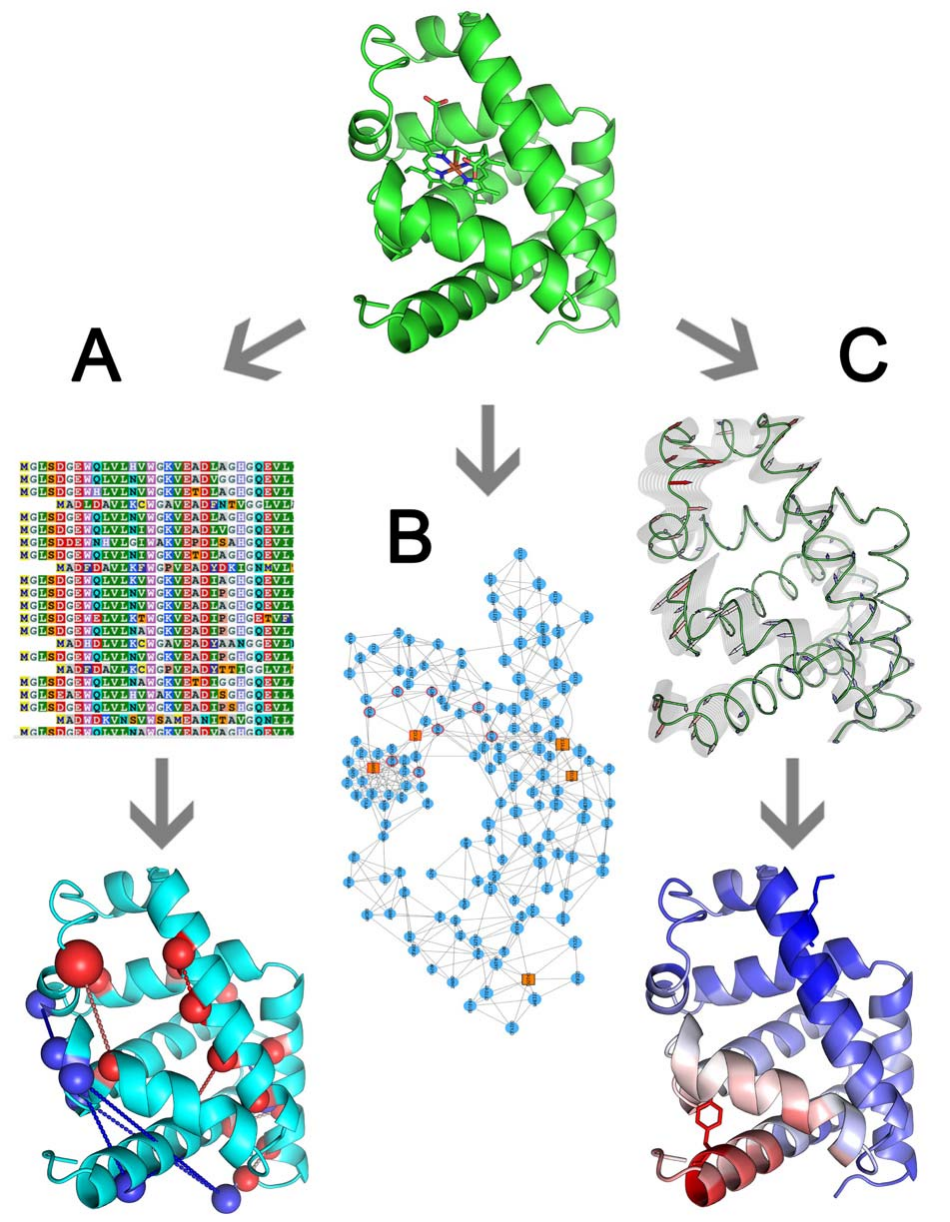
Principles of tools for the prediction of allosteric hotspots. (**A**) Using MSA, correlated pairs of residues are identified and mapped on the protein structure, enabling their division to the pairs in physical contact (red spheres connected by red lines) and remote ones (blue spheres connected by blue lines). (**B**) By analyzing interacting residues, a network representation of the target protein can be derived, from which highly interconnected nodes (orange squares) can be detected. (**C**) Protein dynamics can be estimated through the NMA to outline protein regions that become more rigid (red) or flexible (blue) upon ligand binding.

Analysis of correlated or co-evolving residues has been the tradition in protein structure modeling to predict direct or indirect coupling between pairs or groups of residues [72–74]. This method has been employed to improve the quality of predicted protein structures and protein–protein complexes [75–78]. Identified co-evolving residues have also become a frequent target of engineering aiming at stability [79] and allosteric regulation [80] (Figure 2A).

The visualCMAT web-server is a recent tool focused on the analysis and identification of correlated or co-evolved hotspots [46]. This tool uses an MSA and 3D structure to assess the correlation between residues based on mutual information. Since prediction quality is dependent on the quality of the MSA, the server recommends an integrated Mustguseal web-server [81] for the preparation of the MSA, which reduces the minimal input to a PDB file. A particular limitation is that the server can evaluate only one chain at a time. As a result, correlated or co-evolved residues are identified and mapped onto the structure. These residues are then differentiated into two categories based on a predefined distance cutoff: physically interacting residues and long-range interactions. These long-range interactions may be indicative of allosteric pathways that would be difficult to identify without evolutionary information. Additionally, the server can perform a binding site prediction by identifying pockets with Fpocket [82] and ranking them by the number of present correlated residues. Outputs comprise the list of correlated pairs of positions with the corresponding statistics, the cumulative statistics enumerating involvement of a given position in all pairs found, and their structural visualization. As a test case, the authors predicted correlated residues for the FecA protein from the Porins superfamily [46]. They showed that mutations in some of the identified residues notably altered the transport function of the protein. Owing to a large number of proposed hotspots, the experimental data for validation were available only for a small fraction of the hotspots, excluding some of the highest-ranking ones [46]. Therefore, the lack of data leaves the full extent of visualCMAT applicability to be established yet.

An alternative approach to identify allosteric interactions without the need for a high-quality MSA is to consider a protein structure as a graph of residue–residue interactions (Figure 2B). In a nutshell, protein residues are converted to nodes, often positioned on their Cα atoms, and the edges among these nodes are drawn based on distance cutoffs representing various inter-residue interactions [83]. Finally, the network topology can be analyzed to reveal structurally and functionally relevant connections among residues. Many tools like NetworkAnalyzer [84] or RINerator [85] have been developed for graph-based analyses using different and often incompatible file-formats to store their results, restricting further analyses of generated graphs to specific software, e.g. RINalyzer [85]. To provide a bridge between the most common formats, a user-friendly PDB2Graph toolbox has been developed [47], which, however, depends on proprietary Matlab software. The tool produces an undirected, coarse-grained, distance-based graph that can be exported to different graph formats including Cytoscape [86], Pajek [87] and UCINET [88]. Furthermore, various centrality indices identifying residues critical for protein structure and function can be calculated. Calmodulin, phage T4 lysozyme, Barnase and Ribonuclease HI were used as test cases to assess the applicability of the PDB2Graph tool [47]. Many experimental mutations coincided with the residues identified based on centrality indices, suggesting this method can indeed be of service to protein engineers. Unfortunately, the sparsity of the experimental data did not allow systematic validation of the tool, similarly to visualCMAT.

Taking into consideration a protein not only as a network of connected nodes but also considering protein dynamics (Figure 2C), the structurally identified essential residues (STRESS) tool aims at disclosing allosteric hotspots on the protein surface as well as in its interior [48]. For the surface hotspots, the STRESS tool employs a modified binding leverage approach [89], which was previously implemented in the SPACER web-server [90]. This method combines Monte Carlo (MC)-based ligand docking with normal mode analysis (NMA), which is a computational approach that approximates the local dynamics of a system by a harmonic motion. Outlining the principle of the STRESS method, a simplified representation of a ligand consisting of four-beads is used as a probe to identify putative binding sites on the protein surface. Then, the deformability of these sites is predicted from the 10 lowest frequency normal modes generated by a coarse-grained representation (Cα atoms) NMA provided by the Molecular Modeling Toolkit (MMTK) [91]. Finally, the putative sites are scored and ranked according to their deformability to estimate the degree that the bound ligand would interfere with predicted conformational change. In contrast to SPACER, the STRESS tool markedly reduces a large number of identified putative binding sites by considering all heavy atoms of a protein during docking and applying automatic thresholding. Additionally, STRESS also combines the above described NMA approach with a network analysis in which a protein is represented as a network of interacting residues to expand the scope of analysis to residues critical for communication along a given allosteric pathway (buried allosteric hotspots). Within this network, each edge is weighted depending on the correlation between the movements exhibited by the corresponding interacting residues during NMA. Such a weighted network is subdivided into communities using the Girvan–Newman algorithm [92], and residues critical for interconnection between these communities are detected according to their highest betweenness. For surface hotspot predictions, a list of ranked putative sites with their scores and constituent residues is produced, whereas for interior hotspots, the identity of the critical residues is reported. The applicability of the method was evaluated by its authors on 12 well-studied proteins, in which an average of 55% of known binding sites were identified correctly [48]. Further, the relevance of the identified hotspots was supported by their significantly higher evolutionary conservation in comparison to the non-critical residues calculated on a large dataset of more than 1000 proteins.

Likewise, the AlloSigMA web-server uses NMA aiming not only at the identification of hot-spots but also enabling evaluation of the effects of ligand binding or a mutation on allostery [49] (Figure 2C). This method recognizes four structurally relevant states: (i) unbound/wild type (WT), (ii) bound to a ligand, (iii) mutated and (iv) bound and mutated. In each of these states, allosteric free energy is calculated for each residue using the low-frequency normal modes derived from the Cα-representation of protein provided by the MMTK package and employing a previously developed structure-based statistical mechanical model of allostery [93]. Two scanning approaches can be used in order to detect allosteric hotspots: mutation-based scanning of selected regions or a whole protein, or binding-based scanning with a small probe to triplets of residues. Alternatively, allosteric effects originating from ligand binding, mutation or both combined can be evaluated. It is important to pinpoint that since the method is based on a coarse-grained representation, only two types of mutations can be considered. ‘UP’ mutations, by which the method emulates a mutation to a bulky residue, resulting in stabilizing effect on the local contact network, and ‘DOWN’ mutations, which models alanine/glycine substitutions, resulting in destabilization of the contact network. Aside from interactive visualization, the server provides PDB files with allosteric energies in the B-factor columns. The underlying method was applied to design allosteric mutations aiming at improving the activity of insulin-degrading enzyme toward amyloid β peptide [94]. Out of five constructed single-point mutants, three mutants showed up to 50% increased overall efficiency, while the other two mutants exhibited decreased efficiency [94]. Clearly, more extensive benchmarking is still required to obtain a robust estimate of the method predictive performance.

Whereas all four tools were successfully applied for the identification of hotspots, their quantitative comparison is prevented by the lack of suitable systematic benchmark datasets. Hence, the differences among tools can only be appreciated from a user perspective, considering their speed, ease-of-use and the nature of delivered results. PDB2Graph is the fastest tool, closely followed by visualCMAT, while STRESS is notably slower (Table 1 and see Supplementary Table 1 available online at https: //academic.oup.com/bib). AlloSigMA is by far the most time-demanding tool evaluated in our review, and its allosteric scanning is restricted to a maximum of 2000 residues per analysis. On the other hand, AlloSigMA is the only tool capable of delivering quantitative evaluation of the effect of the mutation on allostery and is much appreciated by the community (Table 1 and see Supplementary Table 2 available online at https: //academic.oup.com/bib). Regarding user-friendliness, only the STRESS tool does not provide interactive analyses of results, and its installation might turn out to be a bit complicated due to its dependence on somewhat older Python 2 modules. Similarly, readers interested in using PDB2Graph will have to procure proprietary Matlab software or consider using older tools like NetworkAnalyzer or RINerator that rely on the open-source Cytospace platform [86].

### Protein–protein interaction hotspots

Interactions of proteins with other proteins are fundamental characteristics of most biological processes such as substrate recognition, metabolism, signaling, pathogenic recognition, protein activation and inactivation. The involved residues have received much attention describing their potential for disrupting or enhancing activity, gaining knowledge about interactions or guiding protein structure prediction [95,96]. Interface residues were initially identified based on the distance between the interacting partners. Recently, approaches based on the Voronoi diagram tessellation have been used instead. Advantages of the Voronoi diagram include robust descriptions of the curvature and connectivity of these interfaces, allowing an unambiguous definition of the interface boundaries and providing a direct way to calculate the contact area [97,98]. Although several tools applying Voronoi tessellation to interface analysis have been published in recent years [97–101], many of them are obsolete or require a substantial degree of programming expertise.

As a user-friendly alternative focusing on protein–protein interactions, the PPI3D web-server was released [50]. This web-server uses a curated local database to retrieve and perform analyses. PPI3D assesses and differentiates three types of interactions: protein–protein, protein–peptide and domain–domain interactions using the weighted Voronoi tessellation implemented in Voronota [102], the correctness of which have been thoroughly tested on more than 90 000 structures from protein databank and almost 30 000 predicted protein structures of various qualities [102]. Initially, PPI3D uses Voronota to identify inter-atom contacts, which are later grouped into inter-residue contacts. To execute the analysis, the web-server accepts three types of submissions: (i) a single sequence to predict protein and peptide-binding sites, (ii) two sequences to find all possible interactions and (iii) a PDB-ID code to identify all interactions in the entry. Importantly, the search for interfaces is not performed on the query protein alone but employs information on homologs too. The results are summarized in the form of a table containing links to a detailed description of interactions, such as interface residues, their contact area and type of interaction.

**Figure 3:**
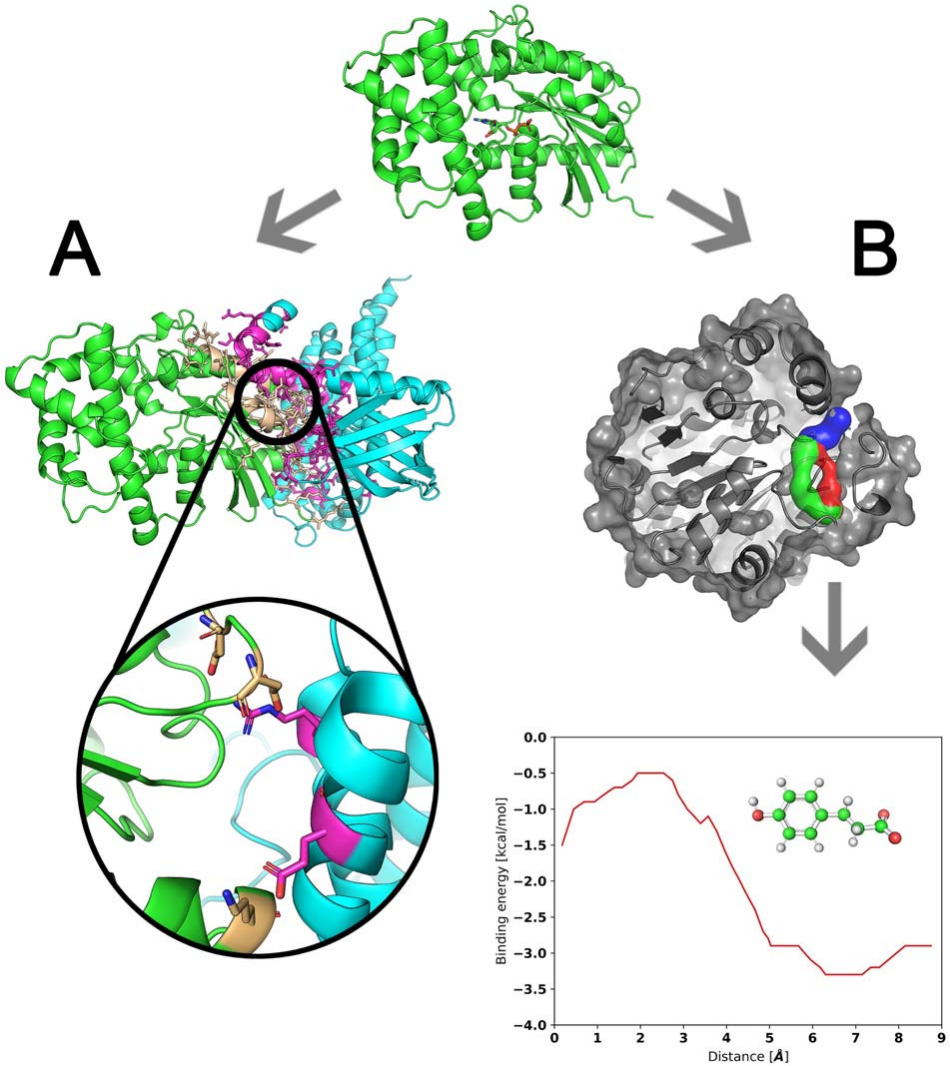
Principles of tools for the prediction of protein interaction hotspots. (**A**) Residues forming interface (gold and pink sticks) of target protein chains (green and cyan cartoon) are analyzed to identify interaction hotspots (gold and pink sticks in the zoomed-in region). (**B**) Transport tunnels (blue, red and green objects) leading from the buried cavity of a target protein to the bulk solvent are delineated and explored by molecular docking of ligand to find protein residues that significantly contribute to the energy barrier of the ligand transport through the tunnel.

When searching for interface hotspots, the objective is typically to improve the binding affinity or avoid mutating those residues rather than destabilizing the protein complex. However, in some cases, disruption is desirable, which is the purpose of the DisruPPI software [51]. To obtain hotspot residues and disruptive mutations, DisruPPI assesses the stability of each monomer and the interactions between them thus enabling disruption of the binding but maintaining or improving the stability of the monomers (Figure 3A). It is important to note that the interface region for analysis must be specified by the user, for which tools like PPI3D can be utilized. Whenever a query sequence is submitted, the software performs a search for homologs in a non-redundant database, the homologs are then aligned, and the interface residues are assessed based on conservation statistics. If the number of homolog sequences is not sufficient, structural modeling is employed to generate likely variants for assessment. In this case, the modeling starts by mutating each residue on the interface to the remaining amino acids, not present in an MSA. Mutations that destabilize the protein according to FoldX [103] or Rosetta [104] are discarded from the candidate pool. With the MSA of homologs, the software selects the most promising candidate sites and takes into consideration their conservation and hydrophobicity. Once candidates are selected, each is tested to obtain disruption and stability scores using a modified INT5 score as the metric [105]. Finally, DisruPPI identifies the lowest energy variant with the highest disruption score. The authors successfully employed the method in three experimental cases: the Hen Egg Lysozyme (HEL) with two anti-HEL antibodies, the HIV-1 glycoprotein gp120 with the cellular CD4 receptor and a red fluorescent protein (RFP) from *Discosoma* sp. These studies resulted in the following: (i) mutations disrupting anti-HEL antibody binding at levels of 27% and 59% of the WT, (ii) the most disruptive mutation of HIV-1-CD4 complex and (iii) five mutations disrupting RFP oligomerization while preserving stable monomers [51].

### Interaction hotspots in ligand-transport tunnels

Residues governing the efficient exchange of cognate ligands between binding sites buried in protein cores and the bulk solvent have recently been recognized as potent hotspots for the engineering of a wide range of properties like activity, selectivity or stability [106]. However, understanding of ligand access and egress pathways is mostly reserved for methods based on molecular dynamics (MD) simulations [107].

CaverDock software was developed as an alternative to expensive analyses of ligand transport using MD simulations [58,59]. It requires pre-computed tunnels for a given macromolecule by software such as CAVER 3.02 [108]. These tunnels are then systematically explored by docking the ligand with a modified AutoDock Vina tool [109]. To automate these calculations, CaverDock has been integrated into a Caver Web 1.0 server [60], which provides automatic calculation of tunnels for a protein of interest that is coupled with follow-up analyses of ligand transport through the user-selected tunnel (Figure 3B). To start a Caver Web 1.0 calculation, the user selects a starting point for tunnel calculation based on detected pockets, the position of ligands, manual selection of residues or Cartesian coordinates. At this stage, detailed information on computed tunnels including their visualization can be accessed. Further, the user can specify tunnel(s) for the examination of transport of ligands, for which the molecule of interest must be uploaded in any format supported by the Open Babel tool [110], provided as an accession code to ZINC15 database [110], or drawn in an interactive window. As a result, the energy profile for each tunnel-compound pair is estimated. The presented functionality of the Caver Web 1.0 platform relating to ligand transport examination is a unique capability compared to alternative tools for tunnel detection [60]. Importantly, the applicability of CaverDock for protein engineering was verified by a detailed computational study of the transport of toxic pollutant 1,2,3-trichloropropane in two variants of haloalkane dehalogenase featuring several advanced MD simulation methods [111]. For this model system, CaverDock was able to pinpoint similar hotspots as the sophisticated MD simulations, confirming its potential for the engineering of ligand transport [111].

## Tools for predicting the effects of mutation

Once hotspots have been selected, the next step is to assess the effects of particular mutations at the site on the target properties of the protein. With this in mind, we have grouped tools based on the type of evaluated feature, starting with protein interactions, followed by flexibility and electrostatics. In this section, we present 10 tools that can be useful for prioritizing particular mutations for inclusion into a smart library based on their predicted effects. MutaBind [52], iSEE [53] and mCSM-PPI2 [54] can be used to estimate the effects of mutation on protein–protein interactions. mCSM-NA [55], PremPDI [56] and mCSM-lig [57] predict mutational effects on interactions of the protein with nucleic acids and ligands. The DynaMut server aims to rapidly evaluate changes in protein dynamics and stability after mutation [61]. The study of modifications in electrostatic potential upon mutation is the domain of the Mutantelec tool [62] and the analysis of electrostatic structures of proteins (AESOP) library [63]. Finally, the BioStructMap integrates data from various sources to help harness the knowledge for the next round of engineering [66]. The majority of the tools discussed in this section rely on machine-learning to derive predictive models from training datasets, while their quantitative performance is evaluated on the testing datasets. To compare performance of tools quantitatively, we summarized Pearson correlation coefficients (PCC) and root-mean-square errors (RMSE) achieved on these datasets as well as the main dataset parameters (Table 2).

**Table 2:** Overview of the datasets employed for training and testing of tools for the prediction of the effect of mutations and the performance of the tools

Tool	Source of data	No. of complexes/proteins	No. of mutations	Pearson correlation coefficient	Root-mean-square error [kcal.mol^−1^]
Training datasets ^a^					
MutaBind	SKEMPI database [112]	80	1925	0.68	1.41
iSEE	DACUM database [113]	57	1102	0.80	1.41
mCSM-PPI2	SKEMPI 2.0 database [114] ^b^	319	8338	0.75	1.30
mCSM-NA	ProNIT database [115]	39	331	0.70	N.A.
PremPDI	ProNIT and dbAMEPNI [116] databases and SAMPDI training dataset [117]	49	219	0.63	0.95
mCSM-lig	Platinum database [118]	>200	763	0.63	2.06
DynaMut	ProTherm database [119] ^b^	131	4594	0.67	1.31
Independent testing datasets					
MutaBind	TA55 from 26th round of the CAPRI [120]	1	1007	0.56	2.58
	TA56 from 26th round of the CAPRI [120]	1	855	0.37	4.27
iSEE	Subset of SKEMPI 2.0 database [114] independent of DACUM database	56	487	0.25	1.32
mCSM-PPI2	TA55 from 26th round of the CAPRI [120]	1	1007	N.A.	2.55
	TA56 from 26th round of the CAPRI [120]	1	855	N.A.	4.06
mCSM-NA	Blind dataset by Barik *et al*. [121]	14	79	0.56 ^c^	N.A.
PremPDI	N.A.	N.A.	N.A.	N.A.	N.A.
mCSM-lig	FluA mutations by Vopel *et al*. [122] ^d^	1	11	0.69	N.A.
DynaMut	PoPMuSiC blind dataset [123] ^b^	67	702	0.70	1.45

^a^Performance of tools on the training datasets was evaluated using leave-one-complex-out cross-validation except for iSEE, mCSM-NA and DynaMut that were evaluated by 10-fold cross-validation instead.

^b^Datasets were enriched with hypothetical reverse mutations.

^c^PCC increases to 0.68 for proximal mutations.

^d^Other testing datasets available but no PCC nor RMSE was reported from their evaluation; N.A., not available.

### Effects of mutations on protein interactions

Proteins interact with each other, nucleic acids or small chemical compounds. As such, they are involved in all essential processes in any living cell. Recognizing the importance of protein–protein interactions, numerous tools have been developed to predict the impact of mutations in residues forming these interactions [124–126].

To expand a set of publicly available methods capable of quantitatively predicting the effects of single-point mutations on binding energy, the MutaBind web-server was developed [52]. This tool uses a consensus of multiple linear regression and a model trained with random forest (RF), both based on 1925 mutations of 80 protein–protein complexes from the SKEMPI database [112] to calculate the changes in binding free energy upon a single-point mutation (∆∆G). The web-server relies on six physicochemical descriptors: Van der Waals interactions, polar solvation energies, unfolding free energies, solvent accessible surface area, conservation score and the ability of proline to introduce constraints on the protein backbone. The prediction performance of the tool was evaluated in a leave-one-complex-out cross-validation, attaining a notable PCC of 0.68, and RMSE of 1.41 kcal.mol^−1^ [52]. Additionally, a separate model was provided for analyses of protease-inhibitor complexes, for which the authors observed improved performance (0.76 PCC and 1.48 kcal.mol^−1^ RMSE) on a subset of their training dataset (862 mutations on 16 complexes) [52]. Two *de novo* designed influenza inhibitors complexed with hemagglutinin used as targets (T55 and T56) in the 26th round of the CAPRI prediction experiment [120] with about 1000 mutations each were employed for independent evaluation. With PCC of 0.56 and 0.37 and RMSE of 2.58 and 4.27 kcal.mol^−1^ for the T55 and T56 targets, respectively [52], MutaBind scored better than three already well-established methods (BeAtMuSiC [126], FoldX [103] and molecular mechanics Poisson-Boltzmann surface area (MM/PBSA) [127]), showing appreciable improvement in PCC by 0.16 over the second best-performing method. The relatively low predictive power might be caused by the use of *de novo* proteins forming an interface that is not commonly present in the training dataset of native proteins, the lack of respective crystal structures and the use of enrichment value as a measurement of binding affinity changes. Also, we would like to highlight that due to the limited number of highly stabilizing mutations present in the SKEMPI and CAPRI datasets, the prediction accuracy for such mutations cannot be adequately estimated and is presumably less reliable. MutaBind web-server can process up to 16 single-point mutations per run, delivering the binding affinity change, confidence level and structure model of each mutant.

A similar approach has been adopted by the iSEE method [53], utilizing an RF approach to evaluate ∆∆G at protein–protein interfaces based on changes in structure-derived solvent accessible surface area, van der Waals, Coulomb and solvation energies and evolutionary information. A subset of the DACUM database [113], consisting of 1102 mutations from 57 protein dimers, was used for method training. In the authors hands, iSEE exhibited 0.80 PCC and RMSE of 1.41 kcal.mol^−1^ during 10-fold cross-validation [53]. Unfortunately, when using a blind dataset of 487 mutations from 56 protein complexes derived from the new SKEMPI 2.0 database [114], iSEE exhibited a PCC of only 0.25 and RMSE of 1.32 kcal.mol^−1^, which is comparable to the performance of the other three state-of-the-art tools evaluated by the authors [53], i.e. FoldX, mCSM [128] and BindProfX [129]. The drop in the ranking performance observed for all four tools is in line with relatively small ∆∆G values present in the dataset, making their evaluation rather sensitive to errors of experimental measurement [53]. Nonetheless, iSEE achieved good correspondence with experimental data on the classification of mutations in the MDM2-p53 complex. It is important to note that iSSE is provided as an R-model only and requires non-trivial data as its input, i.e. the 3D structure of both the WT and mutant complexes, eight energy terms, and evolutionary information in the form of position specific scoring matrix, which represent a marked barrier to widespread application of this tool.

The most recent addition to the toolbox for evaluation of the effects of mutations on protein–protein interaction is the mCSM-PPI2 web-server [54]. A characteristic feature of the mCSM-PPI2 is the use of a graph-based structural signature to represent the environment of the WT residue [128], where the residue environment is described as a graph, with the atoms as nodes and interactions among them as edges. In this scheme, atoms are assigned physicochemical-based pharmacophore types such as hydrophobic, positive, negative, acceptor, donor, aromatic, sulfur and neutral. Aside from the graph-based signatures, seven other types of features are employed in the model: pharmacophore changes due to mutation, structural and sequential residue environment in the WT protein, the nature of the mutant and WT residues, evolutionary information, non-covalent interaction network metrics, energetic terms and atomic fluctuations. Using these features, the mCSM-PPI2 server was trained using the ExtraTrees method on a derivative of the SKEMPI 2.0 database which consists of 4169 experimental variants and their hypothetical reverse mutations from 319 different complexes, giving a total of 8338 single-point mutants. During a leave-one-complex-out cross-validation, the server achieved a PCC of 0.75 and RMSE of 1.30 kcal.mol^−1^ and showed an RMSE of 2.55 and 4.06 kcal.mol^−1^ for mutations of T55 and T56 targets from the 26th round of CAPRI competition [54]. Based on these results and Kendall scores, mCSM-PPI2 is on par with MutaBind and significantly better than the battery of 25 methods including FoldX, BeAtMuSiC, mCSM and MM/PBSA [54]. The mCSM-PPI2 server provides two modes of operation: (i) evaluating the effects of specific mutations defined by the user or (ii) assessing the mutation effects on the interface region by alanine scanning or saturation mutagenesis. For single-point mutations, the server presents the predicted change in binding affinity together with the visualization of the mutation in an interactive NGL viewer [130]. In the interface evaluation mode, an overview of all identified interfaces is provided, with each interface linked to a results page containing a summary with individual mutants listed and available for further exploration, including hotspot identification.

While there is no single study benchmarking all three reviewed tools for predicting the effect of mutations on protein–protein interactions, the results on the independent datasets from the CAPRI competition (Table 2) indicate that both MutaBind and mCSM-PPI2 are comparably accurate and among the best quantitative predictors available. Regarding iSEE performance, this tool reached similar performance to MutaBind in the recent study [131] performed on the testing dataset of iSEE (Table 2). From the user point of view, mCSM-PPI2 offers a convenient option of automated mutation scanning of whole interfaces, and its results are presented in a far more interactive manner. On the contrary, the iSEE method requires the user to precompute all non-trivial input data manually. As the oldest among reviewed tools for protein–protein interaction analysis, Mutabind is the most established in the community (Table 1 and see Supplementary Table 2 available online at https: //academic.oup.com/bib). Finally, our testing indicates that mCMS-PPI2 can perform the complete computation in the shortest time, followed by Mutabind (Table 1 and see Supplementary Table 1 available online at https: //academic.oup.com/bib).

Similarly to protein–protein interactions, interactions of proteins with nucleic acids are the basis of many crucial cellular processes such as replication, repair, recombination, transcription, translation and gene expression regulation. However, the development of rapid predictive methods has had much less success given a notably different physicochemical nature of these interactions, i.e. the prevalence of polar interactions that are often much harder to model precisely [132], and the limited availability of experimental structures as well as affinity data.

Benefiting from the recent release of high-quality data for protein–nucleic acid interactions in the second version of ProNIT database, the mCSM-NA web-server was developed. The tool uses the previously described graph-based approach, adopting additional descriptors for the atoms of nucleic acids, which are divided into three categories: a phosphate group, sugar and nitrogenous base [55]. From this representation, interactions between the protein and nucleic acid atoms were encoded as the graph-based structural signatures and served as an input for Gaussian process regression to train the predictive model. As a training dataset, the ProNIT database composed of 222 mutations from 28 complexes of protein–dsDNA, 42 mutations on six protein–ssDNA complexes and 67 mutations from five protein–RNA complexes was used [115]. On the whole training dataset, a PCC of 0.70 was achieved during 10-fold cross-validation, constituting a small improvement over its generalist predecessor, the mCSM tool, having PCC of 0.67 [128]. Interestingly, PCCs of 0.54, 0.85 and 0.75 were attained for dsDNA, ssDNA and RNA complexes when considered separately, respectively [55]. The authors also performed a blind test on 79 mutations from 14 protein–RNA complexes from the study by Barik and co-workers [121], in which mCSM-NA achieved a PCC of 0.56, raising to 0.68 when considering only mutations in the proximity of the RNA [55]. The user can submit up to 20 single-point mutations per run to mCSM-NA web-server to obtain the predicted change in binding affinity and the effect on protein stability. Importantly, predictions concerning protein–dsDNA interactions should be approached more cautiously, given their notably lower PCC on the training dataset.

An alternative approach to assess the effect of mutations on protein–DNA interactions was adopted by the PremPDI web-server [56], relying primarily on the interaction energy terms computed with the MM/PBSA method. The web-server uses the FoldX tool to model a structure of mutation, which is then, together with the WT structure, shortly energy minimized with NAMD software [133] using CHARMM36 force field [134]. Minimized complexes are then analyzed with the CHARMM package [135] to calculate differences in polar solvation, Van der Waals and electrostatic interaction energies, the number of hydrogen bonds, solvent accessible surface areas, which are further supplemented by information if the mutation occurs on the protein–DNA interface, the length of the protein chain and the pairwise statistical potential for protein folding obtained from the AAindex database [136]. Subsequently, the predictive model is trained by multiple linear regression on a PremPDI dataset, which was compiled from ProNIT and dbAMEPNI [116] databases and training dataset of SAMPDI tool [117], comprising 219 mutations from 49 protein–DNA complexes. A leave-one-complex-out cross-validation yielded PCC of 0.63 and RMSE of 0.95 kcal.mol^−1^. The authors evaluated the performance of PremPDI, SAMPDI and mCSM-NA tools on subsets of training datasets overlapping among the pairs of tools, showing that on these datasets, PremPDI performs similarly to mCSM-NA and markedly outperforms SAMPDI. The PremPDI server provides an interactive calculation setup, enabling up to 16 single-point mutations to be specified, for which the prediction of the effect of the mutation and mutant 3D structure is generated.

Again, the two reviewed tools exhibited similar predictive performance for the effect of mutations on interactions of proteins with DNA. The main difference comes from the user experience and server applicability. Firstly, mCSM-NA can evaluate complexes containing RNA unlike PremPDI. The machine-learning predictions by mCSM-NA are several-fold faster than the demanding physics-based predictions implemented in PremPDI, especially for larger biomolecular systems (Table 1 and see Supplementary Table 1 available online at https: //academic.oup.com/bib). Additionally, the mCSM-NA web-server offers information on the effect of a mutation on protein stability and interactive online visualization. The overall advantages of the mCSM-NA tool are well reflected in its preferential use by the researchers (Table 1 and see Supplementary Table 2 available online at https: //academic.oup.com/bib).

Due to the importance of protein–ligand binding affinities to drug design and enzymology, their predictions have been the focus of numerous tools, including those employing deep-learning algorithms like DeepDTA [137] or *K*_DEEP_ [138]. However, these methods have not been developed for predicting the impact of single-point mutations on binding affinity.

The recently assembled large-scale database of the effects of mutations on the affinity of protein–ligand complexes, Platinum [118], provided a foundation to develop the mCSM-lig web-server [57] capable of such prediction. This web-server is the last of the series of three tools based on the same graph-based approach that, aside from graph-based descriptors of interactions, employs physicochemical descriptors of the ligand, which are complemented by several other features, including a predicted change in stability, depth of evaluated residues, and experimental affinity of WT protein to the ligand. Two separate models for regression and classification of mutations were derived by using a Gaussian process for predictive regression and RF for classification tasks. For model training, a subset of the Platinum database was used, composed of 763 mutations from more than 200 protein–ligand complexes. During the leave-one-complex-out cross-validation on this dataset, the tool reached a PCC of 0.63 and RMSE of 2.06 kcal.mol^−1^ [57]. Moreover, the authors tested the capability of mCSM-lig to discriminate resistance-mutation profiles of different drugs binding to the same site. Evaluation of the binding of chemotherapeutics Imatinib, Nilotinib and Dasatinib to human ABL-kinase with this tool identified over 75% of resistance-causing mutations correctly [57]. In the same way, mutations affecting the binding of Efacirenz and Rilpivirine to HIV-1 reverse transcriptase were identified with more than 80% sensitivity [57]. Application of mCSM-lig to identify mutations improving the binding affinity of fluorescein was tested on the FluA protein. The tool achieved appreciable PCC of 0.69 with experimental data, indicating an aptness for protein engineering tasks [57]. The mCSM-lig web-server requires a structure of a protein–ligand complex, and the ligand affinity to the WT protein to deliver a prediction of the effect of mutation and the mutant 3D structure.

### Effects of mutations on protein dynamics

Mutations can also affect the dynamics of proteins, altering their flexibility or rigidity [42,139,140]. The effect of mutations on protein dynamics can be studied with MD simulations, a powerful method that is computationally demanding and requires considerable expertise to perform. Therefore, quick and ready-to-use methods such as NMA have been developed as an alternative.

To facilitate the use of NMA for evaluating effects of mutation on protein dynamics and stability, DynaMut web-server [61] employs two NMA tools Bio3D [141] and ENCoM [142]. Protein dynamics is combined with the graph-based signature described for mCSM-PPI2, mCSM-NA and mCSM-lig tools to represent the WT structure, stability evaluation of individual conformations via DUET [143], and structural descriptors of the environment of the mutated residue like solvent accessible surface area, residue depth and secondary structure. Using the RF algorithm, the consensus predictor was trained on a dataset derived from the ProTherm database [119], containing 4594 experimental and hypothetical reverse mutations of 131 proteins. During 10-fold cross-validation on the training dataset, DynaMut achieved a PCC of 0.67 and RMSE of 1.31 kcal.mol^−1^, performance was further confirmed with a blind dataset containing 702 forward and reverse mutations (PCC of 0.70 and RMSE of 1.45 kcal.mol^−1^) [61]. With two types of analysis, the web-server can be used to study the dynamic nature of a protein and the effect that point mutations have on its flexibility. DynaMut provides a detailed comparison of the interactions, flexibility and deformability of residues in the WT and mutant protein. 3D structure of the protein is supplemented by predicted energy change in DynaMut, and presented along with three other methods: mCSM, DUET [143] and SDM [144]. Here we would like to add that NMA is known to have, in some instances, a limited sensitivity to a mutation, unless it is responsible for a substantial conformational change [145].

### Effects of mutations on protein electrostatic potential

The electrostatic potential of proteins can affect protein adsorption, ligand binding or thermodynamic stability of a protein complex [146–150]. Different software packages that calculate the electrostatic potential of proteins are available [151–153]. Apart from tools targeting protein stability such as the pStab web-server [154], there are not many tools that assess the effects of mutations on this vital feature.

To overcome this deficiency, the Mutantelec web-server was developed to enable evaluation of the effects that mutations have on electrostatic potential [62]. Additionally, the effects of phosphorylation of serine, threonine and tyrosine can be assessed, broadening the type of analysis that can be performed. The web-server uses Modeller [155] to optimize mutant structures, PDB2PQR [156] to assign atom parameters and APBS [151] to calculate electrostatic potential. As a result, the difference in electrostatics for each residue is represented as a histogram. Additionally, the web-server allows for download of 3D structures and electrostatic maps to visualize in PyMOL (Schrödinger, LLC., USA, http: //www.pymol.org/) or VMD [157]. The p53 protein was analyzed with Mutantelec to explain the effects of Arg249 mutations shown to inactivate the protein function [62].

Similar intentions have prompted a reimplementation of the AESOP framework [158] into a more accessible Python 2 library and web-server [63]. The AESOP library was shown to be applicable to study of the electrostatic similarity of protein families [159,160], perform an alanine scanning of ionizable amino acids to identify possible electrostatic hotspots [161,162], and assess the effects of single-point mutations on the free energy of association [163,164]. Analogously to the Mutantelect workflow, AESOP makes use of Modeller [165] to generate and optimize mutant structures and PDB2PQR and APBS software packages to calculate electrostatic potentials. The capabilities of this web-server are currently limited to alanine scanning. As a result, the AESOP library and web-server generate PDB files of mutants, information on the energy change caused by the mutations, and several predefined structural visualizations.

### Data integration

Engineering efforts do not inevitably end with characterizing properties of the altered protein since information about the mutants can be stored and further reused. This way, an additional cycle of design can be performed iteratively, combining experimental and computational stages. For more convenient analyses, data coming from different sources can be mapped onto the 3D structure of proteins, and there are web-services capable of mapping conservation [166], coevolution [167], biochemical and biomedical annotations [168]. All those services are, however, limited to displaying only one property at a time.

Hence, the BioStructMap tool was developed [66], allowing users to map any sequence-associated function that returns a numeric value onto a 3D protein structure. The BioStructMap package includes pre-defined functions to analyze data, such as mapping polymorphic hotspots, amino acid propensity scales, Tajima’s D index, nucleoside diversity and customized data aggregation. As an input, the package requires a sequence alignment, a PDB file, and a reference sequence matching the sequence of the PDB. The output includes residue-values, as a Python dictionary, which can be mapped to the PDB file on the B-factor column or returned as a text file. Although simple, the most attractive feature of the BioStructMap package is the capability of customization, the only requirement is that the function should return a numerical value. For instance, the user could use numerical results coming from other tools like MutaBind, AESOP or alanine scanning and map those values onto the 3D structure. The resulting PDB file can be analyzed with PyMOL to detect hotspots for the mapped property. BioStructMap is also offered as a web-server that supports analyses with the pre-defined functions.

## Integrative platforms for protein engineering

The typical engineering workflow often covers a range of structural and evolutionary analyses, predictions of the effects of mutation on protein function and stability, and their visualization. Execution of such analysis requires bioengineers to search for suitable computational tools and transfer data from one tool to another, which is frequently complicated by incompatibilities among these tools. To relieve bioengineers of such hurdles, integrative services gather different methodologies for hotspot identification and mutation analyses and streamline the flow of data into automated workflows.

The HotSpot Wizard web-server is a platform developed with the primary purpose of finding protein engineering hotspots by combining structural and sequential analyses [169]. The second version of the service notably expanded the original focus on functional hotspots by providing access to the other three design strategies: stability by structural flexibility, stability by sequence consensus or correlated hotspots [64]. Functional hotspots are identified based on the protein active site and transport tunnels. At the same time, highly conserved residues that are often indispensable for catalysis are avoided in order not to compromise enzyme function. This method of hotspot selection is recommended when substrate specificity is the target property. Regarding stabilizing hotspots, highly mobile residues are identified by their thermal B-factors. Alternatively, an MSA of homologous proteins can be analyzed to find residues differing from the prevalent variant at any given position in homologous proteins. Both strategies were shown to be useful when a protein with improved thermal stability is desired [170,171]. Lastly, the correlated hotspot approach is based on the MSA that is scrutinized by seven methods for coordinated changes in the sequence and combined to a Z-score based consensus. Considering the vast number of identified correlated hotspots, further analyses using alternative approaches are advisable to obtain more focused predictions, unless the modulation of allostery is the engineering target. The main innovation of the current third version of the platform was the integration of an automated protein structure modeling workflow to overcome the applicability limit of previous versions [65]. Additionally, the Rosetta and FoldX tools can now be utilized for the evaluation of the effect of particular mutations at hotspots of interest. As the input, a protein sequence, PDB file or PDB-ID code is required. Additionally, some of the most influential parameters controlling calculations of integrated tools can be modified in advanced settings. Once the calculations are finished, results for each strategy can be accessed from the navigation panel. Further, the effects of selected mutations can be evaluated, used to develop an optimized smart library and generate nucleotide sequences employing the codon usage of a target organism. Overall, the HotSpot Wizard is a user-friendly and well-established service for hotspot identification and library design that has been available for over 10 years and is kept up to date, with the latest version published in 2018. It has been successfully verified in seven engineering studies reported so far (see Supplementary Table 3 available online at https: //academic.oup.com/bib).

## Conclusions, perspectives and challenges

The reviewed computational tools can provide valuable guidance for protein engineering toward tailoring a wide range of target properties. Notably, the applicability of the protein engineering toolbox has been expanded from targeting traditionally engineered regions of proteins, such as binding sites and transport-pathways, to addressing even more elusive targets including protein dynamics and allostery. Here, most tools resorted to approximating the dynamics by rapid methods based on NMA to minimize overall execution time and required computing resources. However, we expect that other approaches including the geometric-constraints-based method, tCONCOORD [172], or the perturbation-based methods, L-RIP or RIPlig [173], will be integrated as an alternative or used alongside NMA to overcome the intrinsic limitations of each method and to provide complementary insights into the structure–dynamics–function relationships of engineered proteins.

There is a clear trend of protein engineering software becoming more user-friendly, commonly featuring an attractive web-server interface. However, we have noted only a handful of integrative efforts to provide meta-servers where the user can employ different approaches to protein design. Even the current one-stop-shop platforms like HotSpot Wizard do not yet offer comprehensive approaches. In particular, they do not allow analysis of possible interactions of the target protein with other molecules, leaving such burdensome tasks to their users. Therefore, an essential step toward complete workflows is to integrate tools for the prediction of macromolecular binding sites and evaluation of actual protein interactions with cognate binding partners, ultimately creating complete pipelines which will be even more attractive for protein engineers.

Since a significant fraction of the reviewed tools employs various methods of supervised machine learning, the quality of the available datasets for development and testing is of utmost importance. We have seen an example of the role that a more extensive dataset can play for the accuracy of the predictive methods for the effect of a mutation on protein–protein interactions. In that case, the release of the SKEMPI 2.0 database, which more than doubled the number of annotated mutations and almost tripled the diversity in protein–protein complexes, enabled the derivation of more accurate predictive models adopted by the mCSA-PPI2 tool. However, even this database is notably biased by the strategies and research objectives traditionally applied in the protein engineering community [114], for example, there is a prevalence of single-point mutations (most frequently to alanines), the majority of mutations being located at the interface (mostly at its core), and the distribution of observed effects of mutations being shifted toward destabilization. This lack of proportionality hampers the development of more accurate and general predictive methods. The impact of such biases on the development of machine learning models for protein engineering and some necessary steps toward resolving these issues have been discussed elsewhere [174].

Another aspect related to the experimental datasets is their sparsity. For most proteins, information on the effects of mutations is available for a few sites only, and even for those that were mutated, often not all variants were tested. Such situations markedly complicate the performance evaluation of tools for hotspot identification and mutation prioritization. The data sparsity also makes any rigorous comparison among different methods almost impossible since experimental data rarely cover the top hits proposed by individual tools. As a remedy, we suggest turning to datasets from systematic mutagenesis projects like the dataset of 13 massively mutated proteins compiled from the literature and patents [175] and, more recently, datasets originating from deep mutation scanning techniques that provide almost complete mapping of the mutation landscape [176,177]. Also, we would like to highlight another initiative, ProtaBank repository [178], which introduces a standardized format for storing and reporting data from protein engineering studies to facilitate accurate comparisons among them. Its utilization has the potential to overcome some of the challenges discussed here.

**Key Points**

- Computationally supported rational and semi-rational protein engineering constitute widely accepted and efficient approaches. These approaches become more accessible to the broad scientific community due to the development of user-friendly tools, 18 of which are reviewed here.
- These novel tools enable identification of engineering hotspots promising for the modification of protein function based on allosteric communication, interactions with other proteins or ligands.
- Mutations can be evaluated for their impact on protein interactions with other macromolecules, protein dynamics or an electrostatic potential that can be estimated by user-friendly software covered in this review.
- Identification of hotspot residues and prediction of the effect of their mutation can be easily performed via integrative platforms like HotSpot Wizard. This platform was successfully applied in multiple design experiments leading to the identification of enhanced variants showcased here.

## Supplementary Material

Supplementary_Table1_bbaa150

Supplementary_Table2_bbaa150

Supplementary_Table3_bbaa150

## Notes

**Carlos Eduardo Sequeiros-Borja** is a PhD candidate at Laboratory of Biomolecular Interactions and Transport, Department of Gene Expression, Institute of Molecular Biology and Biotechnology, Faculty of Biology, Adam Mickiewicz University and the International Institute of Molecular and Cell Biology in Warsaw, Warsaw, Poland. His current interests are the development of bioinformatics methods and tools to study substrate–enzyme interactions and understand the effect of mutations on protein function.

**Bartłomiej Surpeta** is a PhD candidate at Laboratory of Biomolecular Interactions and Transport, Department of Gene Expression, Institute of Molecular Biology and Biotechnology, Faculty of Biology, Adam Mickiewicz University and the International Institute of Molecular and Cell Biology in Warsaw, Warsaw, Poland. He focuses on the application of molecular dynamics simulations to uncover structure–dynamics–function relationships in proteins and rational protein design.

**Jan Brezovsky** is a professor jointly at Laboratory of Biomolecular Interactions and Transport, Department of Gene Expression, Institute of Molecular Biology and Biotechnology, Faculty of Biology, Adam Mickiewicz University and the International Institute of Molecular and Cell Biology in Warsaw. His group develops structural bioinformatics and molecular dynamics simulations methods and applies them to protein engineering and drug discovery.

## References

[1] Savile CK , Janey JM, Mundorff EC, et al. Biocatalytic asymmetric synthesis of chiral amines from ketones applied to sitagliptin manufacture. Science 2010;329:305–9. 20558668 10.1126/science.1188934

[2] Patel RN . Biocatalysis for synthesis of pharmaceuticals. Bioorg Med Chem 2018;26:1252–74. 28648492 10.1016/j.bmc.2017.05.023

[3] Choi JM , Han SS, Kim HS. Industrial applications of enzyme biocatalysis: current status and future aspects. Biotechnol Adv 2015;33:1443–54. 25747291 10.1016/j.biotechadv.2015.02.014

[4] Aldridge S . Industry backs biocatalysis for greener manufacturing. Nat Biotechnol 2013;31:95–6. 23392497 10.1038/nbt0213-95

[5] Vellard M . The enzyme as drug: application of enzymes as pharmaceuticals. Curr Opin Biotechnol 2003;14:444–50. 12943856 10.1016/s0958-1669(03)00092-2

[6] Himmel ME , Ding SY, Johnson DK, et al. Biomass recalcitrance: engineering plants and enzymes for biofuels production. Science 2007;315:804–7. 17289988 10.1126/science.1137016

[7] Kim J , Campbell AS, de Ávila BEF, et al. Wearable biosensors for healthcare monitoring. Nat Biotechnol 2019;37:389–406. 30804534 10.1038/s41587-019-0045-y PMC8183422

[8] Jackson DA , Symons RH, Berg P. Biochemical method for inserting new genetic information into DNA of simian virus 40: circular SV40 DNA molecules containing lambda phage genes and the galactose operon of Escherichia coli. Proc Natl Acad Sci USA 1972;69:2904–9. 4342968 10.1073/pnas.69.10.2904 PMC389671

[9] Cohen SN , Chang ACY. Recircularization and autonomous replication of a sheared R factor DNA segment in *Escherichia coli* transformants. Proc Natl Acad Sci USA 1973;70:1293–7. 4576014 10.1073/pnas.70.5.1293 PMC433482

[10] Adli M . The CRISPR tool kit for genome editing and beyond. Nat Commun 2018;9:1–13. 29765029 10.1038/s41467-018-04252-2 PMC5953931

[11] Wilkinson AJ , Fersht AR, Blow DM, et al. A large increase in enzyme-substrate affinity by protein engineering. Nature 1984;307:187–8. 6690998 10.1038/307187a0

[12] Wells JA , Powers DB, Bott RR, et al. Designing substrate specificity by protein engineering of electrostatic interactions. Proc Natl Acad Sci USA 1987;84:1219–23. 3547407 10.1073/pnas.84.5.1219 PMC304398

[13] Thomas PG , Russell AJ, Fersht AR. Tailoring the pH dependence of enzyme catalysis using protein engineering. Nature 1985;318:375–6.

[14] Kazlauskas RJ , Bornscheuer UT. Finding better protein engineering strategies. Nat Chem Biol 2009;5:526–9. 19620988 10.1038/nchembio0809-526

[15] Arnold FH . Innovation by evolution: bringing new chemistry to life (Nobel lecture). Angew Chem Int Ed Engl 2019;58:14420–6. 31433107 10.1002/anie.201907729

[16] Lutz S . Beyond directed evolution-semi-rational protein engineering and design. Curr Opin Biotechnol 2010;21:734–43. 20869867 10.1016/j.copbio.2010.08.011 PMC2982887

[17] Sebestova E , Bendl J, Brezovsky J, et al. Computational tools for designing smart libraries. Methods Mol Biol 2014;1179:291–314. 25055786 10.1007/978-1-4939-1053-3_20

[18] Davids T , Schmidt M, Böttcher D, et al. Strategies for the discovery and engineering of enzymes for biocatalysis. Curr Opin Chem Biol 2013;17:215–20. 23523243 10.1016/j.cbpa.2013.02.022

[19] Chaparro-Riggers JF , Polizzi KM, Bommarius AS. Better library design: data-driven protein engineering. Biotechnol J 2007;2:180–91. 17183506 10.1002/biot.200600170

[20] Sinha R , Shukla P. Current trends in protein engineering: updates and progress. Curr Protein Pept Sci 2019;20:398–407. 30451109 10.2174/1389203720666181119120120

[21] Swint-Kruse L . Using evolution to guide protein engineering: the devil IS in the details. Biophys J 2016;111:10–8. 27410729 10.1016/j.bpj.2016.05.030 PMC4945580

[22] Pincus D , Pandey JP, Feder ZA, et al. Engineering allosteric regulation in protein kinases. Sci Signal 2018;11:eaar 3250. 10.1126/scisignal.aar3250 PMC6662207 30401787

[23] Sun MGF , Seo MH, Nim S, et al. Protein engineering by highly parallel screening of computationally designed variants. Sci Adv 2016;2:e1600692. 27453948 10.1126/sciadv.1600692 PMC4956399

[24] Allen BD , Nisthal A, Mayo SL. Experimental library screening demonstrates the successful application of computational protein design to large structural ensembles. Proc Natl Acad Sci USA 2010;107:19838–43. 21045132 10.1073/pnas.1012985107 PMC2993350

[25] Silva DA , Yu S, Ulge UY, et al. De novo design of potent and selective mimics of IL-2 and IL-15. Nature 2019;565:186–91. 30626941 10.1038/s41586-018-0830-7 PMC6521699

[26] Dudek HM , de Gonzalo G, Pazmiño DET, et al. Mapping the substrate binding site of phenylacetone monooxygenase from *Thermobifida fusca* by mutational analysis. Appl Environ Microbiol 2011;77:5730–8. 21724896 10.1128/AEM.00687-11 PMC3165276

[27] Lalonde J . Highly engineered biocatalysts for efficient small molecule pharmaceutical synthesis. Curr Opin Biotechnol 2016;42:152–8. 27261887 10.1016/j.copbio.2016.04.023

[28] Li G , bo WJ, Reetz MT. Biocatalysts for the pharmaceutical industry created by structure-guided directed evolution of stereoselective enzymes. Bioorg Med Chem 2018;26:1241–51. 28693917 10.1016/j.bmc.2017.05.021

[29] Bornscheuer UT , Huisman GW, Kazlauskas RJ, et al. Engineering the third wave of biocatalysis. Nature 2012;485:185–94. 22575958 10.1038/nature11117

[30] Tobin P , Richards D, Callender R, et al. Protein engineering: a new frontier for biological therapeutics. Curr Drug Metab 2014;15:743–56. 25495737 10.2174/1389200216666141208151524 PMC4931902

[31] Dvorak P , Bednar D, Vanacek P, et al. Computer-assisted engineering of hyperstable fibroblast growth factor 2. Biotechnol Bioeng 2018;115:850–62. 29278409 10.1002/bit.26531

[32] Dvorak P , Nikel PI, Damborsky J, et al. Bioremediation 3.0: engineering pollutant-removing bacteria in the times of systemic biology. Biotechnol Adv 2017;35:845–66. 28789939 10.1016/j.biotechadv.2017.08.001

[33] Romero-Rivera A , Garcia-Borràs M, Osuna S. Computational tools for the evaluation of laboratory-engineered biocatalysts. Chem Commun 2017;53:284–97. 10.1039/c6cc06055b PMC5310519 27812570

[34] Ebert MC , Pelletier JN. Computational tools for enzyme improvement: why everyone can – and should – use them. Curr Opin Chem Biol 2017;37:89–96. 28231515 10.1016/j.cbpa.2017.01.021

[35] Damborsky J , Brezovsky J. Computational tools for designing and engineering enzymes. Curr Opin Chem Biol 2014;19:8–16. 24780274 10.1016/j.cbpa.2013.12.003

[36] Frushicheva MP , Mills MJL, Schopf P, et al. Computer aided enzyme design and catalytic concepts. Curr Opin Chem Biol 2014;21:56–62. 24814389 10.1016/j.cbpa.2014.03.022 PMC4149935

[37] Sheik Amamuddy O , Veldman W, Manyumwa C, et al. Integrated computational approaches and tools for allosteric drug discovery. Int J Mol Sci 2020;21:847. 10.3390/ijms21030847 PMC7036869 32013012

[38] Wilding M , Hong N, Spence M, et al. Protein engineering: the potential of remote mutations. Biochem Soc Trans 2019;47:701–11. 30902926 10.1042/BST20180614

[39] Liang Z , Verkhivker GM, Hu G. Integration of network models and evolutionary analysis into high-throughput modeling of protein dynamics and allosteric regulation: theory, tools and applications. Brief Bioinform 2019;21:815–35. 10.1093/bib/bbz029 30911759

[40] Petrović D , Kamerlin SCL. Molecular modeling of conformational dynamics and its role in enzyme evolution. Curr Opin Struct Biol 2018;52:50–7. 30205262 10.1016/j.sbi.2018.08.004

[41] Maria-Solano MA , Serrano-Hervás E, Romero-Rivera A, et al. Role of conformational dynamics in the evolution of novel enzyme function. Chem Commun 2018;54:6622–34. 10.1039/c8cc02426j PMC6009289 29780987

[42] Petrović D , Risso VA, Kamerlin SCL, et al. Conformational dynamics and enzyme evolution. J R Soc Interface 2018;15:20180330. 30021929 10.1098/rsif.2018.0330 PMC6073641

[43] Surpeta B , Sequeiros-Borja CE, Brezovsky J. Dynamics, a powerful component of current and future in silico approaches for protein design and engineering. Int J Mol Sci 2020;21:2713. 10.3390/ijms21082713 PMC7215530 32295283

[44] Musil M , Konegger H, Hon J, et al. Computational design of stable and soluble biocatalysts. ACS Catal 2019;9:1033–54.

[45] Liu Q , Xun G, Feng Y. The state-of-the-art strategies of protein engineering for enzyme stabilization. Biotechnol Adv 2019;37:530–7. 31138425 10.1016/j.biotechadv.2018.10.011

[46] Suplatov DA , Sharapova Y, Timonina D, et al. The visual CMAT: a web-server to select and interpret correlated mutations/co-evolving residues in protein families. J Bioinform Comput Biol 2018;16:1840005. 29361894 10.1142/S021972001840005X

[47] Niknam N , Khakzad H, Arab SS, et al. PDB2Graph: a toolbox for identifying critical amino acids map in proteins based on graph theory. Comput Biol Med 2016;72:151–9. 27043857 10.1016/j.compbiomed.2016.03.012

[48] Clarke D , Sethi A, Li S, et al. Identifying allosteric hotspots with dynamics: application to inter- and intra-species conservation. Structure 2016;24:826–37. 27066750 10.1016/j.str.2016.03.008 PMC4883016

[49] Guarnera E , Tan ZW, Zheng Z, et al. AlloSigMA: allosteric signaling and mutation analysis server. Bioinformatics 2017;33:3996–8. 29106449 10.1093/bioinformatics/btx430

[50] Dapkūnas J , Timinskas A, Olechnovič K, et al. The PPI3D web server for searching, analyzing and modeling protein–protein interactions in the context of 3D structures. Bioinformatics 2016;33:935–7. 10.1093/bioinformatics/btw756 28011769

[51] Choi Y , Furlon JM, Amos RB, et al. DisruPPI: structure-based computational redesign algorithm for protein binding disruption. Bioinformatics 2018;34:i245–53. 29949961 10.1093/bioinformatics/bty274 PMC6022686

[52] Li M , Simonetti FL, Goncearenco A, et al. MutaBind estimates and interprets the effects of sequence variants on protein–protein interactions. Nucleic Acids Res 2016;44:W494–501. 27150810 10.1093/nar/gkw374 PMC4987923

[53] Geng C , Vangone A, Folkers GE, et al. iSEE: interface structure, evolution, and energy-based machine learning predictor of binding affinity changes upon mutations. Proteins 2018;87:110–9. 30417935 10.1002/prot.25630 PMC6587874

[54] Rodrigues CHM , Myung Y, Pires DEV, et al. mCSM-PPI2: predicting the effects of mutations on protein-protein interactions. Nucleic Acids Res 2019;47:W338–44. 31114883 10.1093/nar/gkz383 PMC6602427

[55] Pires DEV , Ascher DB. mCSM-NA. Predicting the effects of mutations on protein-nucleic acids interactions. Nucleic Acids Res 2017;45:W241–6. 28383703 10.1093/nar/gkx236 PMC5570212

[56] Zhang N , Chen Y, Zhao F, et al. PremPDI estimates and interprets the effects of missense mutations on protein-DNA interactions. PLoS Comput Biol 2018;14:e1006615. 30533007 10.1371/journal.pcbi.1006615 PMC6303081

[57] Pires DEV , Blundell TL, Ascher DB. mCSM-lig: quantifying the effects of mutations on protein-small molecule affinity in genetic disease and emergence of drug resistance. Sci Rep 2016;6:29575. 27384129 10.1038/srep29575 PMC4935856

[58] Filipovic J , Vavra O, Plhak J, et al. CaverDock: a novel method for the fast analysis of ligand transport. IEEE/ACM Trans Comput Biol Bioinform 2019;1–1. 10.1109/TCBB.2019.2907492 30932844

[59] Vavra O , Filipovic J, Plhak J, et al. CaverDock: a molecular docking-based tool to analyse ligand transport through protein tunnels and channels. Bioinformatics 2019;35:4986–93. 31077297 10.1093/bioinformatics/btz386

[60] Stourac J , Vavra O, Kokkonen P, et al. Caver Web 1.0: identification of tunnels and channels in proteins and analysis of ligand transport. Nucleic Acids Res 2019;47:W414–22. 31114897 10.1093/nar/gkz378 PMC6602463

[61] Rodrigues CHM , Pires DEV, Ascher DB. DynaMut: predicting the impact of mutations on protein conformation, flexibility and stability. Nucleic Acids Res 2018;46:W350–5. 29718330 10.1093/nar/gky300 PMC6031064

[62] Valdebenito-Maturana B , Reyes-Suarez JA, Henriquez J, et al. Mutantelec: an in Silico mutation simulation platform for comparative electrostatic potential profiling of proteins. J Comput Chem 2017;38:467–74. 28114729 10.1002/jcc.24712

[63] Harrison RES , Mohan RR, Gorham RD, et al. AESOP: a Python library for investigating electrostatics in protein interactions. Biophys J 2017;112:1761–6. 28494947 10.1016/j.bpj.2017.04.005 PMC5425408

[64] Bendl J , Stourac J, Sebestova E, et al. HotSpot Wizard 2.0: automated design of site-specific mutations and smart libraries in protein engineering. Nucleic Acids Res 2016;44:W479–87. 27174934 10.1093/nar/gkw416 PMC4987947

[65] Sumbalova L , Stourac J, Martinek T, et al. HotSpot Wizard 3.0: web server for automated design of mutations and smart libraries based on sequence input information. Nucleic Acids Res 2018;46:W356–62. 29796670 10.1093/nar/gky417 PMC6030891

[66] Guy AJ , Irani V, Richards JS, et al. BioStructMap: a Python tool for integration of protein structure and sequence-based features. Bioinformatics 2018;34:3942–4. 29931276 10.1093/bioinformatics/bty474 PMC6223362

[67] Morley KL , Kazlauskas RJ. Improving enzyme properties: when are closer mutations better? Trends Biotechnol 2005;23:231–7. 15866000 10.1016/j.tibtech.2005.03.005

[68] Pavlova M , Klvana M, Prokop Z, et al. Redesigning dehalogenase access tunnels as a strategy for degrading an anthropogenic substrate. Nat Chem Biol 2009;5:727–33. 19701186 10.1038/nchembio.205

[69] Yu H , Huang H. Engineering proteins for thermostability through rigidifying flexible sites. Biotechnol Adv 2014;32:308–15. 24211474 10.1016/j.biotechadv.2013.10.012

[70] Rivalta I , Sultan MM, Lee NS, et al. Allosteric pathways in imidazole glycerol phosphate synthase. Proc Natl Acad Sci USA 2012;109:E1428–36. 22586084 10.1073/pnas.1120536109 PMC3365145

[71] Borgo B , Havranek JJ. Automated selection of stabilizing mutations in designed and natural proteins. Proc Natl Acad Sci USA 2012;109:1494–9. 22307603 10.1073/pnas.1115172109 PMC3277135

[72] Morcos F , Pagnani A, Lunt B, et al. Direct-coupling analysis of residue coevolution captures native contacts across many protein families. Proc Natl Acad Sci USA 2011;108:E1293–301. 22106262 10.1073/pnas.1111471108 PMC3241805

[73] Morcos F , Schafer NP, Cheng RR, et al. Coevolutionary information, protein folding landscapes, and the thermodynamics of natural selection. Proc Natl Acad Sci USA 2014;111:12408–13. 25114242 10.1073/pnas.1413575111 PMC4151759

[74] Cheng RR , Morcos F, Levine H, et al. Toward rationally redesigning bacterial two-component signaling systems using coevolutionary information. Proc Natl Acad Sci USA 2014;111:E563–71. 24449878 10.1073/pnas.1323734111 PMC3918776

[75] Ovchinnikov S , Kamisetty H, Baker D. Robust and accurate prediction of residue-residue interactions across protein interfaces using evolutionary information. Elife 2014;3:e02030. 24842992 10.7554/eLife.02030 PMC4034769

[76] Hopf TA , Schärfe CPI, Rodrigues JPGLM, et al. Sequence co-evolution gives 3D contacts and structures of protein complexes. Elife 2014;3:e03430. 10.7554/eLife.03430 PMC4360534 25255213

[77] Kamisetty H , Ovchinnikov S, Baker D. Assessing the utility of coevolution-based residue-residue contact predictions in a sequence- and structure-rich era. Proc Natl Acad Sci USA 2013;110:15674–9. 24009338 10.1073/pnas.1314045110 PMC3785744

[78] Jones DT , Buchan DWA, Cozzetto D, et al. PSICOV: precise structural contact prediction using sparse inverse covariance estimation on large multiple sequence alignments. Bioinformatics 2012;28:184–90. 22101153 10.1093/bioinformatics/btr638

[79] Franceus J , Verhaeghe T, Desmet T. Correlated positions in protein evolution and engineering. J Ind Microbiol Biotechnol 2017;44:687–95. 27514664 10.1007/s10295-016-1811-1

[80] Reynolds KA , McLaughlin RN, Ranganathan R. Hot spots for allosteric regulation on protein surfaces. Cell 2011;147:1564–75. 22196731 10.1016/j.cell.2011.10.049 PMC3414429

[81] Suplatov DA , Kopylov KE, Popova NN, et al. Mustguseal: a server for multiple structure-guided sequence alignment of protein families. Bioinformatics 2018;34:1583–5. 29309510 10.1093/bioinformatics/btx831

[82] Le Guilloux V , Schmidtke P, Tuffery P. Fpocket: an open source platform for ligand pocket detection. BMC Bioinformatics 2009;10:168. 19486540 10.1186/1471-2105-10-168 PMC2700099

[83] Di Paola L , De Ruvo M, Paci P, et al. Protein contact networks: an emerging paradigm in chemistry. Chem Rev 2013;113:1598–613. 23186336 10.1021/cr3002356

[84] Assenov Y , Ramírez F, Schelhorn S-E, et al. Computing topological parameters of biological networks. Bioinformatics 2008;24:282–4. 18006545 10.1093/bioinformatics/btm554

[85] Doncheva NT , Klein K, Domingues FS, et al. Analyzing and visualizing residue networks of protein structures. Trends Biochem Sci 2011;36:179–82. 21345680 10.1016/j.tibs.2011.01.002

[86] Shannon P , Markiel A, Ozier O, et al. Cytoscape: a software environment for integrated models of biomolecular interaction networks. Genome Res 2003;13:2498–504. 14597658 10.1101/gr.1239303 PMC403769

[87] Batagelj V , Mrvar A. Pajek—analysis and visualization of large networks. In: Jünger M., Mutzel P (eds). Graph drawing software. Berlin, Heidelberg: Springer, 2004, 77–103.

[88] Borgatti SP , Everett MG, Freeman LC. UCINET 6 for Windows: Software for Social Network Analysis. Harvard, MA: Analytic Technologies, 2002.

[89] Mitternacht S , Berezovsky IN. Binding leverage as a molecular basis for allosteric regulation. PLoS Comput Biol 2011;7:e1002148. 21935347 10.1371/journal.pcbi.1002148 PMC3174156

[90] Goncearenco A , Mitternacht S, Yong T, et al. SPACER: server for predicting allosteric communication and effects of regulation. Nucleic Acids Res 2013;41:W266–72. 23737445 10.1093/nar/gkt460 PMC3692057

[91] Hinsen K . The molecular modeling toolkit: a new approach to molecular simulations. J Comput Chem 2000;21:79–85.

[92] Girvan M , Newman MEJ. Community structure in social and biological networks. Proc Natl Acad Sci USA 2002;99:7821–6. 12060727 10.1073/pnas.122653799 PMC122977

[93] Guarnera E , Berezovsky IN. Structure-based statistical mechanical model accounts for the causality and energetics of allosteric communication. PLoS Comput Biol 2016;12:e1004678. 26939022 10.1371/journal.pcbi.1004678 PMC4777440

[94] Kurochkin IV , Guarnera E, Wong JH, et al. Toward allosterically increased catalytic activity of insulin-degrading enzyme against amyloid peptides. Biochemistry 2017;56:228–39. 27982586 10.1021/acs.biochem.6b00783

[95] Petta I , Lievens S, Libert C, et al. Modulation of protein-protein interactions for the development of novel therapeutics. Mol Ther 2016;24:707–18. 26675501 10.1038/mt.2015.214 PMC4886928

[96] Kawabata T . HOMCOS: an updated server to search and model complex 3D structures. J Struct Funct Genomics 2016;17:83–99. 27522608 10.1007/s10969-016-9208-y PMC5274653

[97] Cazals F , Proust F, Bahadur RP, et al. Revisiting the Voronoi description of protein-protein interfaces. Protein Sci 2006;15:2082–92. 16943442 10.1110/ps.062245906 PMC2242599

[98] Ban Y-EA , Edelsbrunner H, Rudolph J. Interface surfaces for protein-protein complexes. J ACM 2006;53:361–78.

[99] Bernauer J , Bahadur RP, Rodier F, et al. DiMoVo: a Voronoi tessellation-based method for discriminating crystallographic and biological protein-protein interactions. Bioinformatics 2008;24:652–8. 18204058 10.1093/bioinformatics/btn022

[100] Rooklin D , Wang C, Katigbak J, et al. AlphaSpace: fragment-centric topographical mapping to target protein-protein interaction interfaces. J Chem Inf Model 2015;55:1585–99. 26225450 10.1021/acs.jcim.5b00103 PMC4550072

[101] Esque J , Léonard S, de Brevern AG, et al. VLDP web server: a powerful geometric tool for analysing protein structures in their environment. Nucleic Acids Res 2013;41:W373–8. 23761450 10.1093/nar/gkt509 PMC3692094

[102] Olechnovič K , Venclovas Č. Voronota: a fast and reliable tool for computing the vertices of the Voronoi diagram of atomic balls. J Comput Chem 2014;35:672–81. 24523197 10.1002/jcc.23538

[103] Schymkowitz J , Borg J, Stricher F, et al. The FoldX web server: an online force field. Nucleic Acids Res 2005;33:W382–8. 15980494 10.1093/nar/gki387 PMC1160148

[104] Schreiber G , Fleishman SJ. Computational design of protein–protein interactions. Curr Opin Struct Biol 2013;23:903–10. 23993666 10.1016/j.sbi.2013.08.003

[105] Pons C , Talavera D, De La Cruz X, et al. Scoring by intermolecular pairwise propensities of exposed residues (SIPPER): a new efficient potential for protein-protein docking. J Chem Inf Model 2011;51:370–7. 21214199 10.1021/ci100353e

[106] Kokkonen P , Bednar D, Pinto G, et al. Engineering enzyme access tunnels. Biotechnol Adv 2019;107386:37. 10.1016/j.biotechadv.2019.04.008 31026496

[107] Nunes-Alves A , Kokh DB, Wade RC. Recent progress in molecular simulation methods for drug binding kinetics. arXiv 2020; 2002.08983. 10.1016/j.sbi.2020.06.022 32771530

[108] Chovancova E , Pavelka A, Benes P, et al. CAVER 3.0: a tool for the analysis of transport pathways in dynamic protein structures. PLoS Comput Biol 2012;e1002708:8. 10.1371/journal.pcbi.1002708 PMC3475669 23093919

[109] Trott O , Olson AJ. Software news and update AutoDock Vina: improving the speed and accuracy of docking with a new scoring function, efficient optimization, and multithreading. J Comput Chem 2010;31:455–61. 19499576 10.1002/jcc.21334 PMC3041641

[110] O’Boyle NM , Banck M, James CA, et al. Open Babel: an open chemical toolbox. J Chem 2011;3:33. 10.1186/1758-2946-3-33 PMC3198950 21982300

[111] Marques SM , Bednar D, Damborsky J. Computational study of protein-ligand unbinding for enzyme engineering. Front Chem 2019;6:650. 30671430 10.3389/fchem.2018.00650 PMC6331733

[112] Moal IH , Fernández-Recio J. SKEMPI: a structural kinetic and energetic database of mutant protein interactions and its use in empirical models. Bioinformatics 2012;28:2600–7. 22859501 10.1093/bioinformatics/bts489

[113] Geng C , Vangone A, Bonvin AMJJ. Exploring the interplay between experimental methods and the performance of predictors of binding affinity change upon mutations in protein complexes. Protein Eng Des Sel 2016;29:291–9. 27284087 10.1093/protein/gzw020

[114] Jankauskaite J , Jiménez-García B, Dapkunas J, et al. SKEMPI 2.0: An updated benchmark of changes in protein-protein binding energy, kinetics and thermodynamics upon mutation. Bioinformatics 2019;35:462–9. 30020414 10.1093/bioinformatics/bty635 PMC6361233

[115] Prabakaran P , An J, Gromiha MM, et al. Thermodynamic database for protein-nucleic acid interactions (ProNIT). Bioinformatics 2001;17:1027–34. 11724731 10.1093/bioinformatics/17.11.1027

[116] Liu L , Xiong Y, Gao H, et al. dbAMEPNI: a database of alanine mutagenic effects for protein-nucleic acid interactions. Database 2018 2018;bay 034. 10.1093/database/bay034 PMC5887268 29688380

[117] Peng Y , Sun L, Jia Z, et al. Predicting protein-DNA binding free energy change upon missense mutations using modified MM/PBSA approach: SAMPDI webserver. Bioinformatics 2018;34:779–86. 29091991 10.1093/bioinformatics/btx698 PMC6048991

[118] Pires DEV , Blundell TL, Ascher DB. Platinum: a database of experimentally measured effects of mutations on structurally defined protein-ligand complexes. Nucleic Acids Res 2015;43:D387–91. 25324307 10.1093/nar/gku966 PMC4384026

[119] Gromiha MM , An J, Kono H, et al. ProTherm: thermodynamic database for proteins and mutants. Nucleic Acids Res 1999;27:286–8. 9847203 10.1093/nar/27.1.286 PMC148158

[120] Janin J , Henrick K, Moult J, et al. CAPRI: a critical assessment of PRedicted interactions. Proteins Struct Funct Gen 2003;52:2–9. 10.1002/prot.10381 12784359

[121] Barik A , Nithin C, Karampudi NBR, et al. Probing binding hot spots at protein-RNA recognition sites. Nucleic Acids Res 2015;44:e9. 26365245 10.1093/nar/gkv876 PMC4737170

[122] Vopel S , Mühlbach H, Skerra A. Rational engineering of a fluorescein-binding anticalin for improved ligand affinity. Biol Chem 2005;386:1097–104. 16307475 10.1515/BC.2005.126

[123] Dehouck Y , Grosfils A, Folch B, et al. Fast and accurate predictions of protein stability changes upon mutations using statistical potentials and neural networks: PoPMuSiC-2.0. Bioinformatics 2009;25:2537–43. 19654118 10.1093/bioinformatics/btp445

[124] Brender JR , Zhang Y. Predicting the effect of mutations on protein-protein binding interactions through structure-based Interface profiles. PLoS Comput Biol 2015;11:e1004494. 26506533 10.1371/journal.pcbi.1004494 PMC4624718

[125] Krüger DM , Gohlke H. DrugScorePPI webserver: fast and accurate in silico alanine scanning for scoring protein-protein interactions. Nucleic Acids Res 2010;38:W480–6. 20511591 10.1093/nar/gkq471 PMC2896140

[126] Dehouck Y , Kwasigroch JM, Rooman M, et al. BeAtMuSiC: prediction of changes in protein-protein binding affinity on mutations. Nucleic Acids Res 2013;41:W333–9. 23723246 10.1093/nar/gkt450 PMC3692068

[127] Kollman PA , Massova I, Reyes C, et al. Calculating structures and free energies of complex molecules: combining molecular mechanics and continuum models. Acc Chem Res 2000;33:889–97. 11123888 10.1021/ar000033j

[128] Pires DEV , Ascher DB, Blundell TL. mCSM: predicting the effects of mutations in proteins using graph-based signatures. Bioinformatics 2014;30:335–42. 24281696 10.1093/bioinformatics/btt691 PMC3904523

[129] Xiong P , Zhang C, Zheng W, et al. BindProfX: assessing mutation-induced binding affinity change by protein Interface profiles with pseudo-counts. J Mol Biol 2017;429:426–34. 27899282 10.1016/j.jmb.2016.11.022 PMC5963940

[130] Rose AS , Bradley AR, Valasatava Y, et al. NGL viewer: web-based molecular graphics for large complexes. Bioinformatics 2018;34:3755–8. 29850778 10.1093/bioinformatics/bty419 PMC6198858

[131] Zhang N , Chen Y, Lu H, et al. MutaBind2: predicting the impacts of single and multiple mutations on protein-protein interactions. iScience 2020;23:100939. 32169820 10.1016/j.isci.2020.100939 PMC7068639

[132] Stranges PB , Kuhlman B. A comparison of successful and failed protein interface designs highlights the challenges of designing buried hydrogen bonds. Protein Sci 2013;22:74–82. 23139141 10.1002/pro.2187 PMC3575862

[133] Phillips JC , Braun R, Wang W, et al. Scalable molecular dynamics with NAMD. J Comput Chem 2005;26:1781–802. 16222654 10.1002/jcc.20289 PMC2486339

[134] Mac Kerell AD , Bashford D, Bellott M, et al. All-atom empirical potential for molecular modeling and dynamics studies of proteins. J Phys Chem B 1998;102:3586–616. 24889800 10.1021/jp973084f

[135] Brooks BR , Brooks CL, Mackerell AD, et al. CHARMM: the biomolecular simulation program. J Comput Chem 2009;30:1545–614. 19444816 10.1002/jcc.21287 PMC2810661

[136] Kawashima S , Pokarowski P, Pokarowska M, et al. AAindex: amino acid index database, progress report 2008. Nucleic Acids Res 2008;36:D202–5. 17998252 10.1093/nar/gkm998 PMC2238890

[137] Öztürk H , Özgür A, Ozkirimli E, et al. DeepDTA: deep drug-target binding affinity prediction. Bioinformatics 2018;34:i821–9. 30423097 10.1093/bioinformatics/bty593 PMC6129291

[138] Jiménez J , Škalič M, Martínez-Rosell G, et al. KDEEP: protein-ligand absolute binding affinity prediction via 3D-convolutional neural networks. J Chem Inf Model 2018;58:287–96. 29309725 10.1021/acs.jcim.7b00650

[139] Gagné D , French RL, Narayanan C, et al. Perturbation of the conformational dynamics of an active-site loop alters enzyme activity. Structure 2015;23:2256–66. 26655472 10.1016/j.str.2015.10.011 PMC4680847

[140] Otten R , Liu L, Kenner LR, et al. Rescue of conformational dynamics in enzyme catalysis by directed evolution. Nat Commun 2018;9:1314. 29615624 10.1038/s41467-018-03562-9 PMC5883053

[141] Grant BJ , Rodrigues APC, ElSawy KM, et al. Bio3d: an R package for the comparative analysis of protein structures. Bioinformatics 2006;22:2695–6. 16940322 10.1093/bioinformatics/btl461

[142] Frappier V , Najmanovich RJ. A coarse-grained elastic network atom contact model and its use in the simulation of protein dynamics and the prediction of the effect of mutations. PLoS Comput Biol 2014;10:e1003569. 24762569 10.1371/journal.pcbi.1003569 PMC3998880

[143] Pires DEV , Ascher DB, Blundell TL. DUET: a server for predicting effects of mutations on protein stability using an integrated computational approach. Nucleic Acids Res 2014;42:W314–9. 24829462 10.1093/nar/gku411 PMC4086143

[144] Pandurangan AP , Ochoa-Montaño B, Ascher DB, et al. SDM: a server for predicting effects of mutations on protein stability. Nucleic Acids Res 2017;45:W229–35. 28525590 10.1093/nar/gkx439 PMC5793720

[145] Bauer JA , Pavlović J, Bauerová-Hlinková V. Normal mode analysis as a routine part of a structural investigation. Molecules 2019;24:3293. 10.3390/molecules24183293 PMC6767145 31510014

[146] McCammon JA . Darwinian biophysics: electrostatics and evolution in the kinetics of molecular binding. Proc Natl Acad Sci USA 2009;106:7683–4. 19416830 10.1073/pnas.0902767106 PMC2683110

[147] Kawano F , Suzuki H, Furuya A, et al. Engineered pairs of distinct photoswitches for optogenetic control of cellular proteins. Nat Commun 2015;6:6256. 25708714 10.1038/ncomms7256

[148] Borgia A , Borgia MB, Bugge K, et al. Extreme disorder in an ultrahigh-affinity protein complex. Nature 2018;555:61–6. 29466338 10.1038/nature25762 PMC6264893

[149] Pakulska MM , Donaghue IE, Obermeyer JM, et al. Encapsulation-free controlled release: electrostatic adsorption eliminates the need for protein encapsulation in PLGA nanoparticles. Sci Adv 2016;e1600519:2. 10.1126/sciadv.1600519 PMC4928928 27386554

[150] Contessoto VG , de Oliveira VM, Fernandes BR, et al. TKSA-MC: a web server for rational mutation through the optimization of protein charge interactions. Proteins 2018;86:1184–8. 30218467 10.1002/prot.25599

[151] Baker NA , Sept D, Joseph S, et al. Electrostatics of nanosystems: application to microtubules and the ribosome. Proc Natl Acad Sci USA 2001;98:10037–41. 11517324 10.1073/pnas.181342398 PMC56910

[152] Walsh I , Minervini G, Corazza A, et al. Bluues Server: electrostatic properties of wild-type and mutated protein structures. Bioinformatics 2012;28:2189–90. 22711791 10.1093/bioinformatics/bts343

[153] Rocchia W , Alexov E, Honig B. Extending the applicability of the nonlinear Poisson-Boltzmann equation: multiple dielectric constants and multivalent ions. J Phys Chem B 2001;105:6507–14.

[154] Gopi S , Devanshu D, Krishna P, et al. pStab: prediction of stable mutants, unfolding curves, stability maps and protein electrostatic frustration. Bioinformatics 2018;34:875–7. 29092002 10.1093/bioinformatics/btx697 PMC6049017

[155] Šali A , Blundell TL. Comparative protein modelling by satisfaction of spatial restraints. J Mol Biol 1993;234:779–815. 8254673 10.1006/jmbi.1993.1626

[156] Dolinsky TJ , Czodrowski P, Li H, et al. PDB2PQR: expanding and upgrading automated preparation of biomolecular structures for molecular simulations. Nucleic Acids Res 2007;35:W522–5. 17488841 10.1093/nar/gkm276 PMC1933214

[157] Humphrey W , Dalke A, Schulten K. VMD: visual molecular dynamics. J Mol Graph 1996;14:33–8. 8744570 10.1016/0263-7855(96)00018-5

[158] Kieslich CA , Morikis D, Yang J, et al. Automated computational framework for the analysis of electrostatic similarities of proteins. Biotechnol Prog 2011;27:316–25. 21485028 10.1002/btpr.541

[159] López de Victoria A , Kieslich CA, Rizos AK, et al. Clustering of HIV-1 subtypes based on gp 120 V3 loop electrostatic properties. BMC Biophys 2012;5:3. 22313935 10.1186/2046-1682-5-3 PMC3295656

[160] Kieslich CA , Morikis D. The two sides of complement C3d: evolution of electrostatics in a link between innate and adaptive immunity. PLoS Comput Biol 2012;8:e1002840. 23300422 10.1371/journal.pcbi.1002840 PMC3531323

[161] Harrison ESR , Gorham RD, Morikis D. Energetic evaluation of binding modes in the C3d and factor H (CCP 19-20) complex. Protein Sci 2015;24:789–802. 25628052 10.1002/pro.2650 PMC4420527

[162] Gorham RD , Rodriguez W, Morikis D. Molecular analysis of the interaction between staphylococcal virulence factor Sbi-IV and complement C3d. Biophys J 2014;106:1164–73. 24606940 10.1016/j.bpj.2014.01.033 PMC4026783

[163] Mohan RR , Gorham RD, Morikis D. A theoretical view of the C3d: CR2 binding controversy. Mol Immunol 2015;64:112–22. 25433434 10.1016/j.molimm.2014.11.006

[164] Liu Y , Kieslich CA, Morikis D, et al. Engineering pre-SUMO4 as efficient substrate of SENP2. Protein Eng Des Sel 2014;27:117–26. 24671712 10.1093/protein/gzu004 PMC3966678

[165] Fiser A , Do RKG, Šali A. Modeling of loops in protein structures. Protein Sci 2000;9:1753–73. 11045621 10.1110/ps.9.9.1753 PMC2144714

[166] Ashkenazy H , Erez E, Martz E, et al. ConSurf 2010: calculating evolutionary conservation in sequence and structure of proteins and nucleic acids. Nucleic Acids Res 2010;38:W529–33. 20478830 10.1093/nar/gkq399 PMC2896094

[167] Baker FN , Porollo A. CoeViz: a web-based tool for coevolution analysis of protein residues. BMC Bioinformatics 2016;17:119. 26956673 10.1186/s12859-016-0975-z PMC4782369

[168] Segura J , Sanchez-Garcia R, Martinez M, et al. 3DBIONOTES v2.0: a web server for the automatic annotation of macromolecular structures. Bioinformatics 2017;33:3655–7. 28961691 10.1093/bioinformatics/btx483 PMC5870569

[169] Pavelka A , Chovancova E, Damborsky J. HotSpot Wizard: a web server for identification of hot spots in protein engineering. Nucleic Acids Res 2009;37:W376–83. 19465397 10.1093/nar/gkp410 PMC2703904

[170] Reetz MT , Carballeira JD, Vogel A. Iterative saturation mutagenesis on the basis of b factors as a strategy for increasing protein thermostability. Angew Chem Int Ed Engl 2006;45:7745–51. 17075931 10.1002/anie.200602795

[171] Amin N , Liu AD, Ramer S, et al. Construction of stabilized proteins by combinatorial consensus mutagenesis. Protein Eng Des Sel 2004;17:787–93. 15574484 10.1093/protein/gzh091

[172] Seeliger D , Haas J, de Groot BL. Geometry-based sampling of conformational transitions in proteins. Structure 2007; 15:1482–92 17997973 10.1016/j.str.2007.09.017

[173] Kokh DB , Czodrowski P, Rippmann F, et al. Perturbation approaches for exploring protein binding site flexibility to predict transient binding pockets. J Chem Theory Comput 2016;12:4100–13. 27399277 10.1021/acs.jctc.6b00101

[174] Mazurenko S , Prokop Z, Damborský J. Machine learning in enzyme engineering. ACS Catal 2019;10:1210–23.

[175] Bendl J , Stourac J, Salanda O, et al. PredictSNP: robust and accurate consensus classifier for prediction of disease-related mutations. PLoS Comput Biol 2014;10:e1003440. 24453961 10.1371/journal.pcbi.1003440 PMC3894168

[176] Gupta K , Varadarajan R. Insights into protein structure, stability and function from saturation mutagenesis. Curr Opin Struct Biol 2018;50:117–25. 29505936 10.1016/j.sbi.2018.02.006 PMC6078801

[177] Fowler DM , Fields S. Deep mutational scanning: a new style of protein science. Nat Methods 2014;11:801–7. 25075907 10.1038/nmeth.3027 PMC4410700

[178] Wang CY , Chang PM, Ary ML, et al. ProtaBank: a repository for protein design and engineering data. Protein Sci 2018;27:1113–24. 29575358 10.1002/pro.3406 PMC5980626

