# Supplementary material for: Recent advances in user-friendly computational tools to engineer protein function
Source: Brief Bioinform. 2020 Jul 31;22(3):bbaa150. doi: 10.1093/bib/bbaa150 (PMC8138880; doi:10.1093/bib/bbaa150)
Supplement: Supplementary_Table1_bbaa150 [file supplementary_table1_bbaa150.docx]

**Supplementary Table 1. Approximate runtimes of reviewed tools.**

| **Tool** | **Small protein** | | |  | | **Large protein** | | | | **Evaluated type of analysis** | |
| --- | --- | --- | --- | --- | --- | --- | --- | --- | --- | --- | --- |
|  | **PDB-ID** | **No. of residues** | **Runtime [minutes] ^a^** |  | **PDB-ID** | | **No. of residues** | **Runtime [minutes] ^a^** |  | |  |
| VisualCMAT | 1MWC | 153 | < 1 |  | 5XC7 | | 451 | 6 | Default | |  |
| PDB2Graph | 1MWC | 153 | 1 |  | 5XC7 | | 451 | 3 | CA contact graph | |  |
| STRESS | 1MWC | 153 | 22 |  | 5XC7 | | 451 | 50 | Surface and Interior critical residue identification | |  |
| AlloSigMA | 1MWC | 153 | 170 |  | 5XC7 | | 451 | 440 | Allosteric signaling map | |  |
| PPI3D | 4KML | 241 | < 1 min |  | 4IWS | | 518 | < 1 | PDB entry search | |  |
| DisruPPI | N.A. | N.A. | N.A. |  | N.A. | | N.A. | N.A. | N.A. | |  |
| MutaBind | 4KML | 241 | 15 |  | 4IWS | | 518 | 37 | Mutation | |  |
| iSEE | 1YCR | 109 | < 1 ^b^ |  | N.A. | | N.A. | N.A. | Example | |  |
| mCSM-PPI2 | 4KML | 241 | < 1 |  | 4IWS | | 518 | 7 | Mutation | |  |
| mCSM-NA | 5OND | 155 | < 1 |  | 4TYN | | 509 | < 1 | Mutation | |  |
| PremPDI | 5OND | 155 | 9 |  | 4TYN | | 509 | 27 | Mutation | |  |
| mCSM-lig | 4J8P | 159 | 2 |  | 1E71 | | 501 | 4 | Mutation | |  |
| CaverDock | 1MWC | 153 | 2 |  | 5XC7 | | 451 | 3 | Ligand transport analysis | |  |
| DynaMut | 1MWC | 153 | 5 |  | 5XC7 | | 451 | 12 | Mutation effects prediction | |  |
| Mutantelec | 1MWC | 153 | 7 |  | 5XC7 | | 451 | 24 | Site-specific | |  |
| AESOP ^c^ | 1MWC | 153 | 1 |  | 5XC7 | | 451 | 4 | Mutation | |  |
| HotSpot Wizard | 1MWC | 153 | 3 |  | 5XC7 | | 451 | 5 | Default | |  |
| BioStructMap | 1MWC | 153 | < 1 |  | 5XC7 | | 451 | 2 | Tajima D mapping | |  |

^a^ – Runtimes for stand-alone tools were evaluated on a notebook with the following parameters: Intel Core i7-5500U 2.40GHz, 8GB RAM, Ubuntu 18.04.4 LTS, using a single core; ^b^ – The runtime is reported for an example case for which non-trivial input data has already been precomputed; ^c^ – supports parallelism on multiple cores
