# Supplementary material for: Recent advances in user-friendly computational tools to engineer protein function
Source: Brief Bioinform. 2020 Jul 31;22(3):bbaa150. doi: 10.1093/bib/bbaa150 (PMC8138880; doi:10.1093/bib/bbaa150)
Supplement: Supplementary_Table2_bbaa150 [file supplementary_table2_bbaa150.docx]

**Supplementary Table 2. Overview of utilization of the reviewed tools by the scientific community.**

| **Tool** | **Publication year** | **Total peer-reviewed citations ^a^** | **Total practical applications ^b^** | **Engineering usage ^c^** | **Usage for protein annotation** | **Reviews and books** | **Other predictive tools** | **Others citations ^d^** |
| --- | --- | --- | --- | --- | --- | --- | --- | --- |
| VisualCMAT | 2018 | 6 | 0 | 0 | 0 | 2 | 1 | 3 |
| PDB2Graph | 2016 | 1 | 0 | 0 | 0 | 0 | 0 | 1 |
| STRESS | 2016 | 29 | 2 | 0 | 2 | 15 | 2 | 10 |
| AlloSigMA | 2017 | 36 | 13 | 12 | 1 | 14 | 4 | 5 |
| PPI3D | 2016 | 13 | 2 | 0 | 2 | 3 | 0 | 8 |
| DisruPPI | 2018 | 3 | 0 | 0 | 0 | 1 | 0 | 2 |
| MutaBind | 2016 | 55 | 15 | 15 | 0 | 20 | 9 | 11 |
| iSEE | 2018 | 12 | 1 | 0 | 1 | 3 | 6 | 2 |
| mCSM-PPI2 | 2019 | 7 | 1 | 1 | 0 | 1 | 4 | 1 |
| mCSM-NA | 2017 | 27 | 5 | 5 | 0 | 9 | 11 | 2 |
| PremPDI | 2018 | 3 | 0 | 0 | 0 | 2 | 1 | 0 |
| mCSM-lig | 2016 | 48 | 20 | 20 | 0 | 11 | 12 | 5 |
| CaverDock | 2019 | 9 | 4 | 1 | 3 | 4 | 0 | 1 |
| DynaMut | 2018 | 68 | 43 | 41 | 2 | 9 | 8 | 8 |
| Mutantelec | 2017 | 2 | 2 | 2 | 0 | 0 | 0 | 0 |
| AESOP | 2017 | 6 | 6 | 6 | 0 | 0 | 0 | 0 |
| HotSpot Wizard | 2016 | 60 | 21 | 18 | 3 | 20 | 3 | 16 |
| BioStructMap | 2018 | 0 | 0 | 0 | 0 | 0 | 0 | 0 |

^a^ – according to Google Scholar on May 20, 2020; ^b^ – the overall number of citations to studies using the tools for practical purposes (= the sum of Intended usage and usage for protein annotation); ^c^ – originally intended application of the tool like assessing the effect of mutation or identifying hotspots; ^d^ – citations related to original observations in the paper introducing the reviewed tools or acknowledging the method’s existence, etc.
