# Supplementary material for: Recent advances in user-friendly computational tools to engineer protein function
Source: Brief Bioinform. 2020 Jul 31;22(3):bbaa150. doi: 10.1093/bib/bbaa150 (PMC8138880; doi:10.1093/bib/bbaa150)
Supplement: Supplementary_Table3_bbaa150 [file supplementary_table3_bbaa150.docx]

**Supplementary Table 3. Successful examples of HotSpot Wizard application to guide protein engineering projects.**

| **HotSpot Wizard** | **Target property** | **Protein** | **Property of improved protein** | **Reference** |
| --- | --- | --- | --- | --- |
| 2.0 | catalysis | computationally designed formolase | catalytic efficiency improved by up to 72 % | [1] |
|  |  | β-*N*-acetylhexosaminidases from *Actinomycetales* and *Bacteroidetes* sp. | transglycosylation yield increased up to 9-fold | [2] |
|  |  | laccase from *Fusarium oxysporum* | catalytic efficiency improved by up to 10-fold | [3] |
|  | stability | xylanase from *Talaromyces leycettanus* | melting temperature increased by up to 7.8°C | [4] |
|  | specificity | nucleoside deoxyribosyltransferase II from *Lactobacillus helveticus* | up to 5.2‑fold higher conversion rate of 2’,3’-dideoxyinosine | [5] |
| 3.0 | stability | L-rhamnose isomerase from *Caldicellulosiruptor obsidiansis* OB47 | melting temperature increased by 3°C; activity half-lives at 70 and 80°C increased by 7.7 and 1.1 h, respectively | [6] |
|  |  | levansucrase from *Brenneria* sp. | melting temperature increased by up to 2.8°C, and the half-lives at 35, 45 and 55°C were increased by was increased by 11.7-, 2.9-, and 2.4-fold, respectively | [7] |

**Supplementary References:**

1. Zhang L, Singh R, Sivakumar D, et al. An artificial synthetic pathway for acetoin, 2,3-butanediol, and 2-butanol production from ethanol using cell free multi-enzyme catalysis. Green Chem. 2018; 20:230–242

2. Jamek SB, Muschiol J, Holck J, et al. Loop Protein Engineering for Improved Transglycosylation Activity of a β‐ *N* ‐Acetylhexosaminidase. ChemBioChem 2018; 19:1858–1865

3. Kwiatos N, Jędrzejczak-Krzepkowska M, Krzemińska A, et al. Evolved Fusarium oxysporum laccase expressed in Saccharomyces cerevisiae. Sci. Rep. 2020; 10:1–11

4. Wang X, Ma R, Xie X, et al. Thermostability improvement of a Talaromyces leycettanus xylanase by rational protein engineering. Sci. Rep. 2017; 7:1–9

5. Li J, Yu L, Li J, et al. Establishment of a high throughput-screening system for nucleoside deoxyribosyltransferase II mutant enzymes with altered substrate specificity. J. Biosci. Bioeng. 2019; 128:22–27

6. Chen Z, Chen J, Zhang W, et al. Improving Thermostability and Catalytic Behavior of l -Rhamnose Isomerase from Caldicellulosiruptor obsidiansis OB47 toward d -Allulose by Site-Directed Mutagenesis. J. Agric. Food Chem. 2018; 66:12017–12024

7. Xu W, Peng J, Zhang W, et al. Enhancement of the Brenneria sp. levansucrase thermostability by site-directed mutagenesis at Glu404 located at the “-TEAP-” residue motif. J. Biotechnol. 2019; 290:1–9
